# Scaling up fact-checking using the wisdom of crowds

**DOI:** 10.1126/sciadv.abf4393

**Published:** 2021-09-01

**Authors:** Jennifer Allen, Antonio A. Arechar, Gordon Pennycook, David G. Rand

**Affiliations:** 1 Sloan School of Management, Massachusetts Institute of Technology, Cambridge, MA, USA.; 2 Center for Research and Teaching in Economics, CIDE, Aguascalientes, Mexico.; 3 Centre for Decision Research and Experimental Economics, CeDEx, Nottingham, UK.; 4 Hill/Levene Schools of Business, University of Regina, Regina, Canada.; 5 Institute for Data, Systems, and Society, Massachusetts Institute of Technology, Cambridge, MA, USA.; 6 Department of Brain and Cognitive Sciences, Massachusetts Institute of Technology, Cambridge, MA, USA.

## Abstract

Professional fact-checking, a prominent approach to combating misinformation, does not scale easily. Furthermore, some distrust fact-checkers because of alleged liberal bias. We explore a solution to these problems: using politically balanced groups of laypeople to identify misinformation at scale. Examining 207 news articles flagged for fact-checking by Facebook algorithms, we compare accuracy ratings of three professional fact-checkers who researched each article to those of 1128 Americans from Amazon Mechanical Turk who rated each article’s headline and lede. The average ratings of small, politically balanced crowds of laypeople (i) correlate with the average fact-checker ratings as well as the fact-checkers’ ratings correlate with each other and (ii) predict whether the majority of fact-checkers rated a headline as “true” with high accuracy. Furthermore, cognitive reflection, political knowledge, and Democratic Party preference are positively related to agreement with fact-checkers, and identifying each headline’s publisher leads to a small increase in agreement with fact-checkers.

## Teaser

When rating articles’ accuracy, a small politically balanced crowd of laypeople yields high agreement with fact-checkers.

## INTRODUCTION

The spread of misinformation on social media—including blatantly false political “fake news,” misleading hyper-partisan news, and other forms of inaccurate content—has become a major matter of societal concern and focus of academic research in recent years (*1*). In particular, there is a great deal of interest in understanding what can be done to reduce the reach of online misinformation. One of the most prominent approaches to combating misinformation, which technology companies such as Facebook and Twitter are currently using (*2*, *3*) and which has received considerable attention within academia [for a review, see (*4*)], is the use of professional fact-checkers to identify and label false or misleading claims.

Fact-checking has the potential to greatly reduce the proliferation and impact of misinformation in at least two different ways. First, fact-checking can be used to inform users about inaccuracies. Debunking false claims typically reduces incorrect belief (*5*) [early concerns about potential “backfire effects” (*6*, *7*) have not been supported by subsequent work; for a review, see (*8*)]. In the context of social media specifically, putting warning labels on content that has been contested by fact-checkers substantially reduces sharing intentions (*9*–*11*). Second, social media platforms can use fact-checking to influence the likelihood that particular pieces of content are shown to users. Using ranking algorithms to demote content that is contested by fact-checkers can markedly reduce the number of users who are exposed (*12*).

As it is typically implemented, however, fact-checking has substantial problems with both scalability and trust and therefore falls far short of realizing its potential. Professional fact-checking is a laborious process that cannot possibly keep pace with the enormous amount of content posted on social media every day. This lack of coverage not only markedly reduces the impact of corrections but also has the potential to increase belief in, and sharing of, misinformation that fails to get checked via the “implied truth effect”: People may infer that lack of warning implies that a claim has been verified (*10*). Furthermore, even when fact-check warnings are successfully applied to misinformation, their impact may be reduced by lack of trust. For example, according to a Poynter study, 70% of Republicans and 50% of Americans overall think that fact-checkers are biased and distrust fact-checking corrections (*13*).

Here, we investigate a potential solution to both of these problems: harnessing the “wisdom of crowds” (*14*, *15*) to make fact-checking scalable and protect it from allegations of bias. Unlike professional fact-checkers, who are in short supply, it is easy (and inexpensive) to recruit large numbers of laypeople to rate headlines, thereby allowing scalability. By recruiting laypeople from across the political spectrum, it is easy to create layperson ratings that are politically balanced and thus cannot be accused of having liberal bias.

But would such layperson ratings actually generate useful insight into the accuracy of the content being rated? Professional fact-checkers have a great deal of training and expertise that enables them to assess information quality (*16*). Conversely, the American public has a remarkably low level of media literacy. For example, in a 2018 Pew poll, a majority of Americans could not even reliably distinguish factual statements from opinions (*17*). Furthermore, layperson judgments may be unduly influenced by partisanship and politically motivated reasoning. Thus, there is reason to be concerned that laypeople would not be effective in classifying the accuracy of news articles.

This, however, is where the power of the wisdom of crowds comes into play. A large literature shows that even if the ratings of individual laypeople are noisy and ineffective, aggregating their responses can lead to highly accurate crowd judgments. For example, the judgment of a diverse, independent group of laypeople has been found to outperform the judgment of a single expert across a variety of domains, including guessing tasks, medical diagnoses, and predictions of corporate earnings (*14*, *15*, *18*, *19*).

Most closely related to the current paper, crowd ratings of the trustworthiness of news publishers were very highly correlated with the ratings of professional fact-checkers (*20*, *21*). These publisher-level ratings, however, may have limited utility for fighting online misinformation. First, there is a great deal of heterogeneity in the quality of content published by a given outlet (*22*). Thus, using publisher-level ratings may lead to a substantial number of false negatives and false positives. In other words, publisher-level ratings are too coarse to reliably classify article accuracy. This interferes with the effectiveness of both labeling and down-ranking problematic content. Second, publisher trust ratings are largely driven by familiarity: People overwhelmingly distrust news publishers that they are unfamiliar with (*20*, *21*). Thus, using publisher-level ratings unfairly punishes publishers that produce accurate content but are either new or niche outlets. This is highly problematic given that much of the promise of the internet and social media as positive societal forces comes from reducing the barrier to entry for new and specialized content producers.

To address these shortfalls and increase the utility of fact-checking, we ask whether the wisdom of crowds is sufficiently powerful to allow laypeople to successfully tackle the substantially harder, and much more practically useful, problem of rating the veracity of individual articles. Previous efforts at using the crowd to identify misinformation have typically focused on allowing users to flag content that they encounter on platform and believe is problematic, and then algorithmically leveraging this user flagging activity (*23*). Such approaches, however, are vulnerable to manipulation: Hostile actors can engage in coordinated attacks—potentially using bots—to flood the reporting system with misleading responses, for example, engaging in coordinated flagging of actually accurate information that is counter to their political ideology. However, this danger is largely eliminated by using a rating system in which random users are invited to provide their opinions about a specific piece of content (as in, for example, election polling), or simply hiring laypeople to rate content (as is done with content moderation). When the crowd is recruited in this manner, it is much more difficult for the mechanism to be infiltrated by a coordinated attack, as the attackers would have to be invited in large numbers to participate and suspicious accounts could be screened out when selecting which users to invite to rate content.

Thus, in contrast to most previous work on misinformation identification, we explore a crowdsourcing approach in which participants are recruited from an online labor market and asked to rate randomly selected articles. We investigate how well laypeople perform when making judgments based on reading only the headline and the lede of the article rather than reading the full article and/or conducting their own research into the article’s veracity. We do so for two reasons. First, we are focused on scalability and thus are seeking to identify an approach that involves a minimum amount of time per rating. Directly rating headlines and ledes is much quicker than reading full articles and doing research. Second, this approach protects against articles that have inaccurate or sensational headlines but accurate texts. Given that most users do not read past the headline of articles on social media (*24*), it is the accuracy of the headline (rather than the full article) that is most important for preventing exposure to misinformation online. Last, in an effort to optimize crowd performance, we also explore the impact of (i) identifying the article’s publisher [via domain of the uniform resource locator (URL), e.g., breitbart.com (*22*)], which could either be informative or work to magnify partisan bias, and (ii) selecting layperson raters based on individual difference characteristics (*25*, *26*) that we find to be correlated with truth discernment.

A particular strength of our approach involves stimulus selection. Past research has demonstrated that laypeople can discern truth from falsehoods on stimulus sets curated by experimenters (*25*, *27*); for example, Bhuiyan *et al.* (*28*) found that laypeople ratings were highly correlated with expert ratings for claims about scientific topics that had a “high degree of consensus among domain experts, as opposed to political topics in which the potential for stable ground truth is much more challenging.” However, the crowd’s performance will obviously depend on the particular statements that participants are asked to evaluate. For example, discerning the truth of “the earth is a cube” versus “the earth is round” will lead to a different result from “the maximum depth of the Pacific Ocean is 34,000 feet” versus “the maximum depth of the Pacific Ocean is 36,000 feet.” In each case, the first statement is false while the second statement is true, but the first comparison is far easier than the second. Thus, when evaluating how well crowds can help scale up fact-checking programs, it is essential to use a set of statements that—unlike past work—accurately represent the misinformation detection problem facing social media companies. To that end, Facebook provided us with a set of articles for evaluation. The articles were sampled from content posted on Facebook in an automated fashion to upsample for content that (i) involved civic topics or health-related information, (ii) was predicted by Facebook’s internal models to be more likely to be false or misleading (using signals such as comments on posts expressing disbelief, false news reports, and pages that have shared misinformation in the past), and/or (iii) was widely shared (i.e., viral). Using these articles, we can directly assess the crowd’s ability to fact-check a set of potentially problematic content that is representative of what social media platforms would have directed to professional fact-checkers.

In our experiment, we recruited 1128 American laypeople from Amazon Mechanical Turk. Each participant was presented with the headline and lede of 20 articles (sampled randomly from a full set of 207 article URLs); half of the participants were also shown the domain of the article’s publisher (i.e., the source). Participants rated each article on seven dimensions related to accuracy, which we averaged to construct an aggregate accuracy rating (Cronbach’s α = 0.96), as well as providing a categorical rating of true, misleading, false, or can’t tell. We then compared the layperson headline + lede ratings to ratings generated by three professional fact-checkers doing detailed research on the veracity of each article. For further details, see Methods.

## RESULTS

To provide a baseline for evaluating layperson performance, we begin by assessing the level of agreement among the professional fact-checkers. The average correlation across articles between the three fact-checkers’ aggregate accuracy ratings was *r* = 0.62 (range = 0.53 to 0.81, *P* < 0.001). Considering the categorical ratings, all three fact-checkers gave the same rating for 49.3% of articles, two of three fact-checkers gave the same rating for 42.0% of articles, and all three fact-checkers gave different ratings for 8.7% of articles. The Fleiss κ for the categorical ratings is 0.38 (*P* < 0.001).

On the one hand, these results demonstrate considerable agreement among the fact-checkers: The aggregate accuracy rating correlation is quite high, and at least two of three fact-checkers’ categorical ratings agreed for over 90% of the articles. At the same time, however, the aggregate accuracy rating correlation is far from 1, and the categorical ratings’ κ statistic only indicates “fair” agreement. This disagreement among fact-checkers is not unique to the fact-checkers we recruited for this study. For example, a study comparing the scores given by FactCheck.org and PolitiFact using ordinal rating scales found a correlation of *r* = 0.66 (*29*), while another study found inter–fact-checker agreement as low as 0.47 when comparing the ratings of well-known fact-checking organizations (*30*). Furthermore, as described in section S9, ratings generated by a set of four professional journalists fact-checking the same set of articles used in our study had an average correlation of *r* = 0.67.

This level of variation in the fact-checker ratings has important implications for fact-checking programs, such as emphasizing the importance of not relying on ratings from just a single fact-checker for certifying veracity and highlighting that “truth” is often not a simple black-and-white classification problem. Moreover, this is an even larger issue for political news: Among the 109 political URLs, the correlation among the fact-checkers was only *r* = 0.56, compared to *r* = 0.69 among the 98 nonpolitical URLs. With this in mind, we use the variation among experts as a benchmark against which to judge the performance of the layperson ratings, to which we now turn.

**Fig. 1.**
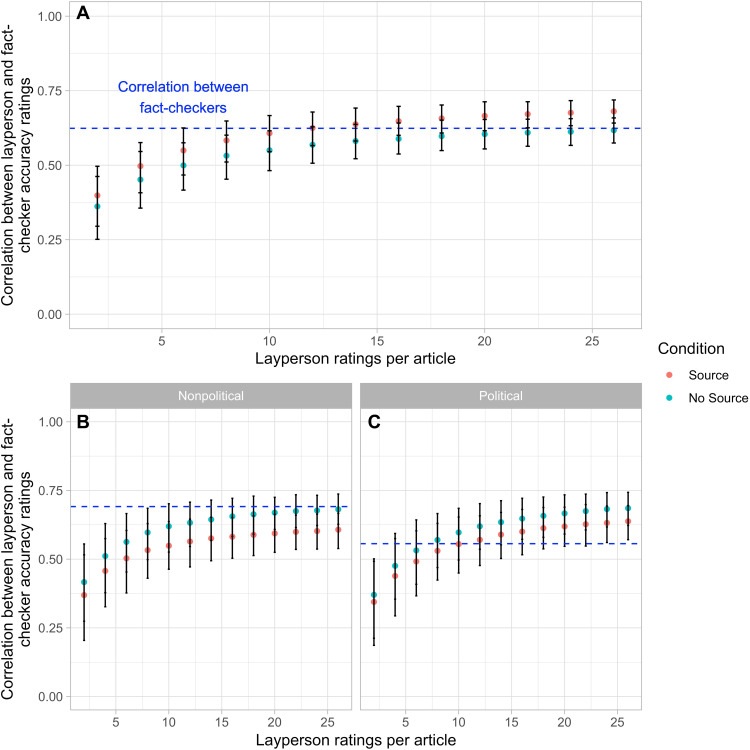
Correlation across articles between (i) politically balanced layperson aggregate accuracy ratings based on reading the headline and lede and (ii) average fact-checker research-based aggregate accuracy ratings, as a function of the number of layperson ratings per article. Laypeople are grouped by condition (Source versus No Source). For results showing up to 100 laypeople per article, see fig. S7; for results using the average of the correlations with a single fact-checker rather than the correlation with the average of the fact-checker ratings, see fig. S9. Panels show results for (**A**) all articles, (**B**) nonpolitical articles, and (**C**) political articles. The dashed line indicates the average Pearson correlation between fact-checkers (all articles, *r* = 0.62; nonpolitical articles, *r* = 0.69; political articles, *r* = 0.56). Error bars indicate 95% confidence intervals.

In our first analysis, we examine how the correlation between the layperson aggregate accuracy ratings and the fact-checker aggregate accuracy ratings varies with the size of the crowd [i.e., the number of layperson ratings per article, *k*, as smaller—and thus more scalable—crowds can often approximate the performance of larger crowds (*31*–*33*)] (see Fig. 1A). We begin by comparing the Source and No-Source conditions. We find that the correlation between the laypeople and the fact-checkers is consistently higher in the Source condition, although the difference is comparatively small (increase of between 0.03 and 0.06, Pearson’s *r*, for the correlation) and only becomes statistically significant for higher values of *k* (*P* < 0.05 for *k* ≥ 24).

To assess how well the layperson judgments correlate with the fact-checkers’ in absolute terms, we use the correlation between the fact-checker ratings as a benchmark (*34*). Perhaps surprisingly, even with a small number of layperson ratings per article, the correlation between the laypeople and the fact-checkers is not significantly lower than the correlation among the fact-checkers. Specifically, starting at *k* = 8 for the Source condition and *k* = 12 for the No-Source condition, we find that the correlation between laypeople and the fact-checkers does not significantly differ from the average correlation between the fact-checkers [Source condition: *k* = 8, *r* = 0.58, 95% confidence interval (CI) = 0.51 to 0.65, *P* = 0.20; No-Source condition: *k* = 12, *r* = 0.57, 95% CI = 0.50 to 0.63, *P* = 0.08]. Furthermore, in the Source condition, the correlation between the laypeople and the fact-checkers actually gets significantly higher than the correlation between the fact-checkers once *k* becomes sufficiently large (*k* = 22, *r* = 0.66, 95% CI = 0.61 to 0.71; *P* < 0.05 for all *k* ≥ 22). Examining political versus nonpolitical URLs separately (Fig. 1, B and C), we see that the layperson judgments agree with the fact-checkers to the same extent for both types of news. Together with the observation that there is substantially less agreement among the fact-checkers for political news, this means that the crowd performs particularly well relative to the intra–fact-checker benchmark for political news. Our results therefore indicate that a relatively small number of laypeople can produce an aggregate judgment, given only the headline and lede of an article, that approximates the judgments of professional fact-checkers—particularly for political headlines.

The advantage of these analyses is that they provide an apples-to-apples comparison between laypeople and fact-checkers by using aggregate accuracy ratings for both groups. In practice, however, it is often desirable to draw actionable conclusions about whether or not any particular article is true, which these analyses do not. To this end, our second analysis approach uses the layperson aggregate accuracy ratings to build a classifier that determines whether or not each article is true. To assess the performance of this classifier, we compare its classification with the modal fact-checker categorical rating (an article is labeled “1” if the modal fact-checker response is “True” and “0” otherwise; see section S5 for analysis where an article is labeled “1” if the modal fact-checker response is “False” and “0” otherwise). We then evaluate the area under the curve (AUC), which measures accuracy while accounting for differences in base rates and is a standard measure of model performance in fields such as machine learning (*35*).

The estimate of the AUC when considering all articles asymptotes with a crowd of around 26 at 0.86 for the Source condition and 0.83 for the No-Source condition (*k* = 26; Source condition: AUC = 0.86, 95% CI = 0.84 to 0.87; No-Source condition, AUC = 0.83, 95% CI = 0.81 to 0.85). This can be interpreted as meaning that when shown a randomly selected true article and a randomly selected not-true (i.e., false, misleading, or can’t tell) article with source information, a crowd of 26 laypeople will rate the true article higher than the not-true article 86% of the time.

**Fig. 2.**
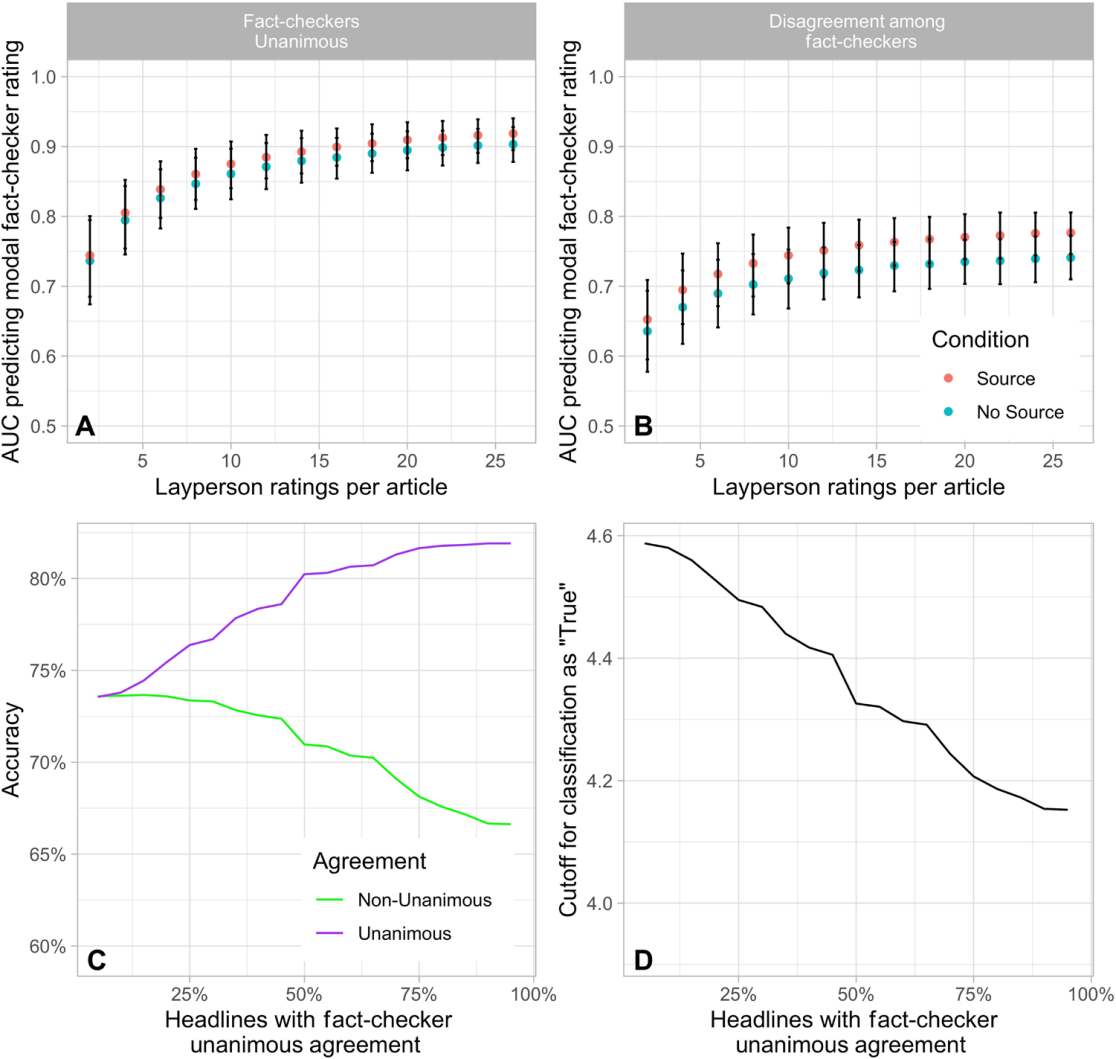
Classifying articles as true versus non-true based on layperson aggregate Likert ratings. (**A** and **B**) AUC scores as a function of the number of layperson ratings per article and source condition. AUC is calculated using a model in which the average layperson aggregate Likert rating is used to predict the modal fact-checker categorical rating, where the fact-checker rating is coded as “1” if the modal rating is “True” and “0” otherwise. Error bars indicate 95% confidence intervals. For full receiver operating characteristics curves using a politically balanced crowd of size 26, see section S14. (**C**) Out-of-sample accuracy for ratings from a politically-balanced crowd of size 26 given source information, calculated separately for unanimous and non-unanimous headlines, as the proportion of unanimous headlines in the sample increases. (**D**) Cutoff for classifying an article as “True” as the proportion of unanimous headlines in the sample increases.

This overall AUC analysis, however, does not take into account heterogeneity across articles in the level of fact-checker agreement. Pragmatically, we should expect—and normatively we should demand—less predictive power from layperson judgments for articles where there was disagreement among the fact-checkers. The AUC is substantially higher when considering the articles in which there was a consensus among the three fact-checkers (102 articles, asymptoting with an AUC of 0.90 to 0.92; Fig. 2A) compared to the articles for which there was disagreement among the fact-checkers (105 articles, asymptoting at AUC of 0.74 to 0.78; Fig. 2B). Thus, the layperson ratings do a very good job of classifying the articles for which there is a relatively clear correct answer and do worse on articles that fact-checkers also struggle to classify. While of course it is a priori impossible to tell which articles will generate consensus or disagreement among fact-checkers, these results suggest that perfect crowd prediction might even be impossible (given the sort of headlines that are flagged for fact-checking by Facebook) due to limitations of the fact-checkers’ agreement and difficulty of the task. It is also encouraging to note that despite the lower levels of consensus among fact-checkers for political versus nonpolitical headlines, the AUC performance between the crowd is not significantly worse for political articles compared to nonpolitical articles (see section S6).

We further explore the ability of crowd ratings to classify articles by simulating how the accuracy of the layperson ratings varies based on the share of headlines with unanimous fact-checker agreement in the stimulus set. Crucially, in these analyses, the threshold used for classifying an article as true must be the same for unanimous versus nonunanimous headlines (given that in real applications, fact-checker unanimity will not be known ex ante); for simplicity, we focus on the Source condition in these analyses. As expected, we find that as the share of unanimous articles increases, the accuracy of the crowd increases, going from an out-of-sample accuracy of 74.2% with a set of all nonunanimous articles to 82.6% with a set of all unanimous articles (Fig. 2C). Although the optimal aggregate accuracy scale cutoff decreases as the share of unanimous headlines increases, it remains within a fairly restricted range (Fig. 2D). Regardless of stimulus make-up, the optimal model is somewhat conservative, in that the average crowd rating must be slightly above the aggregate accuracy scale midpoint for an article to be rated as true.

**Fig. 3.**
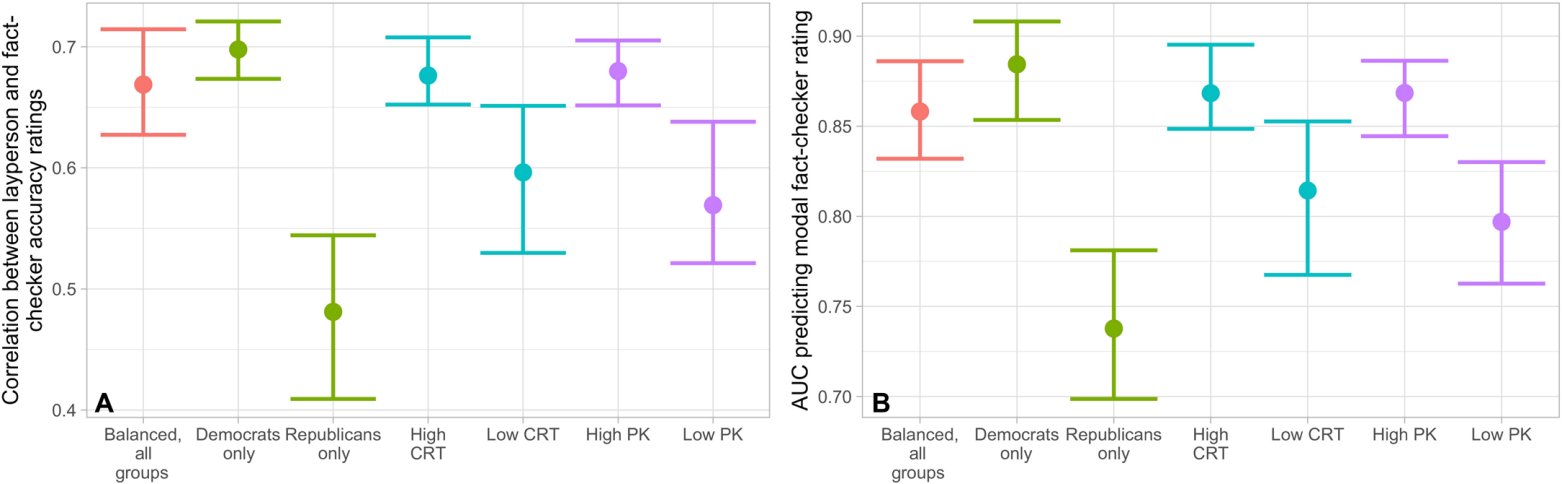
Comparing crowds with different layperson compositions to a baseline, politically balanced crowd. (**A**) Pearson correlations between the average aggregate accuracy rating of a crowd of size 26 and the average aggregate accuracy rating of the fact-checkers. (**B**) AUC for the average aggregate accuracy rating of a crowd of size 26 predicting whether the modal fact-checker categorical rating is true. For both (A) and (B), we compare the baseline to a crowd of only Democrats versus only Republicans, a politically balanced crowd of participants who scored above the median on the CRT versus at or below the median on the CRT, and a politically balanced crowd of participants who score above the median on political knowledge versus at or below the median on political knowledge. Means and CIs are generated using bootstraps with 1000 iterations (see section S2 for details). For analysis comparing political to nonpolitical headlines, see section S13.

Last, we examine how individual differences among laypeople relate to agreement with fact-checker ratings, and whether it is possible to substantially improve the performance of the crowd by altering its composition. In particular, we focus on three individual differences that have been previously associated with truth discernment (*27*): partisanship, political knowledge, and cognitive reflection (the tendency to engage in analytic thinking rather than relying on intuition). For each individual difference, we collapse across Source and No-Source conditions, and begin by fixing a crowd size of *k* = 26 (at which point crowd performance mostly asymptotes). We then examine (i) the correlation between layperson and fact-checker aggregate Likert ratings and (ii) the AUC for predicting whether the fact-checkers’ modal categorical rate is “true” (Fig. 3).

As expected, we see clear differences. Democrats are significantly more aligned with fact-checkers than Republicans (correlation, *P* < 0.001; AUC, *P* < 0.001); participants who are higher on political knowledge are significantly more aligned with fact-checkers than participants who are lower on political knowledge (correlation, *P* < 0.001; AUC, *P* < 0.001); and participants who are higher on cognitive reflection are significantly more aligned with fact-checkers than participants who are lower on cognitive reflection (correlation, *P* = 0.01; AUC, *P* = 0.02). The political knowledge and cognitive reflection results held among both Democrats and Republicans(see section S7). Notably, however, restricting to the better-performing half of participants for each individual difference does not lead to a significant increase in performance over the baseline crowd when examining correlation with fact-checkers’ average aggregate accuracy rating (Democrats versus Baseline: *P* = 0.35; High cognitive reflection test (CRT) versus Baseline: *P* = 0.74; High political knowledge (PK) versus Baseline: *P* = 0.57) or AUC when predicting model fact-checker categorical rating (Democrats versus Baseline: *P* = 0.18; High CRT versus Baseline: *P* = 0.59; High PK versus Baseline: *P* = 0.60). While perhaps surprising, this pattern often arises in wisdom-of-crowds phenomena. The existence of uncorrelated observations from low performers amplifies the high-performer signal by canceling out noise. Thus, while it is important that any given crowd includes some high performers, it is not necessary to exclude low performers to achieve good performance when the crowd is sufficiently large. Using only higher truth-discernment participants does, however, reduce the crowd size needed to reach high agreement with the fact-checkers (see section S8)—that is, crowds that are high on cognitive reflection, political knowledge, or preference for the Democratic Party reach their asymptotic agreement with fact-checkers at substantially smaller crowd sizes. For example, for all three individual differences, restricting to the high performers roughly halves the number of participants needed for the correlation between laypeople and the fact-checkers to reach the average correlation between the fact-checkers. Thus, selecting better-performing raters may boost scalability.

## DISCUSSION

Here, we have shown that crowdsourcing can help identify misinformation at scale. We find that, after judging merely the headline and lede of an article, a small, politically balanced crowd of laypeople can match the performance of fact-checkers researching the full article. Selecting raters whose characteristics make them more likely to agree with the fact-checkers (e.g., more deliberative, higher political knowledge, and more liberal) or providing more information (the article’s publisher) leads to only minimal improvements in the crowd’s agreement with the fact-checkers. This indicates the robustness of the crowd wisdom that we observe and suggests that our results are not particularly sensitive to our specific participant population and task design—and thus that our results are likely to generalize. Together, our findings suggest that crowdsourcing could be a powerful tool for scaling fact-checking on social media.

That these positive results were achieved using small groups of untrained laypeople without research demonstrates the viability of a fact-checking pipeline that incorporates crowdsourcing. Such an approach need not rely on users volunteering their time to provide ratings but could be scalable even with social media platforms compensating raters. For example, in our study, each rating took on average 35.7 s, raters were paid $0.15/min (1.2× greater than the federal minimum wage in the United States), and we achieved good performance with crowds of size 10. Thus, the crowdsourcing approach used here produced useful quality ratings at a cost of roughly $0.90 per article.

Our results also have practical implications for the manner in which crowdsourcing is implemented. In particular, we advocate for using continuous crowdsourced accuracy ratings as a feature in newsfeed ranking, proportionally down-ranking articles according to their scores. A continuous feature incorporates the signal in the crowd’s ratings while guarding against errors that accompany sharp cutoffs of true versus false. In addition, down-ranking has the benefit of lowering the probability that a user encounters misinformation at all, guarding against the illusory truth effect by which familiar falsities seem more true after repetition (*9*, *36*). While corrections to misinformation have generally been shown to be effective (*5*, *37*), that efficacy is dependent on the manner of correction and the possibility of a familiarity backfire effect cannot be ruled out (*9*) [although there has been consistent evidence against it; see (*8*)]. Preventing the spread of misinformation by limiting exposure is a proactive way to fight fake news. Furthermore, work on accuracy prompts/nudges indicates yet another benefit of crowdsourcing: Simply asking users to rate the accuracy of content primes the concept of accuracy and makes them more discerning in their subsequent sharing (*38*, *39*).

The promise of crowd ratings that we observe here does not mean that professional fact-checkers are no longer necessary. Rather, we see crowdsourcing as just one component of a misinformation detection system that incorporates machine learning, layperson ratings, and expert judgments. While machine-learning algorithms are scalable and have been shown to be somewhat effective for detecting fake news, they are also domain specific and thus susceptible to failure in our rapidly changing information environment (*40*–*44*). In addition, the level of disagreement between fact-checkers raises concerns about systems that (i) privilege the unilateral decisions of a single fact-checker or (i) use a single fact-checker’s ratings as “ground truth” in supervised machine-learning models, as is often done [see also (*45*)]. We see the integration of crowdsourced ratings as helping to address the shortcomings of these other methods: Using crowd ratings as training inputs can help artificial intelligence adapt more quickly, and supplement and extend professional fact-checking. Allowing a greater number of articles to be assessed and labeled will directly expand the impact of fact-checking, as well as reducing the chance that unlabeled inaccurate claims will be believed more because they lack a warning (i.e., the “implied truth effect”) (*10*).

In addition to these practical implications for fighting misinformation, our results have substantial theoretical relevance. First, we contribute to the ongoing debate regarding the role of reasoning in susceptibility to misinformation. Some have argued that people engage in “identity protective cognition” (*46*–*49*) such that more reasoning leads to greater polarization rather than greater accuracy. In contrast to this argument, our findings of greater cognitive reflection and political knowledge being associated with higher fact-checker agreement support the “classical reasoning” account whereby reasoning leads to more accurate judgments. In particular, our results extend previous evidence for classical reasoning due to our use of an ecologically valid stimulus set. It is possible that previous evidence of a link between cognitive sophistication and truth discernment (*9*, *20*, *25*, *50*, *51*) was induced by experimenters selecting headlines that were obviously true versus false. Here, we show that the same finding is obtained when using a set of headlines where veracity is much less cut and dried, and which represents an actual sample of misleading content (rather than being hand-picked by researchers). Relatedly, our individual differences results extend previous work on partisan differences in truth discernment, as we find that Republicans show substantially less agreement with professional fact-checkers than Democrats in our naturally occurring article sample—even for nonpolitical articles (see section S13)—although these partisanship results should be interpreted with caution given that our sample was not nationally representative.

Second, our results contribute to the literature on source effects. While a large literature has shown that people preferentially believe information from trusted sources [for a review, see (*52*)], this work has usually focused on information shared by people who are more or less trusted. In contrast, numerous recent studies have examined the impact of the publisher associated with a news headline (either by hiding versus revealing the actual publisher or by experimentally manipulating which publisher is associated with a given article) and have surprisingly found little effect (*22*, *26*, *53*). To explain this lack of effect, it has been theorized that information about the publisher only influences accuracy judgments insomuch as there is a mismatch between publisher trustworthiness and headline plausibility (*22*). In the absence of such a mismatch, publisher information is redundant. However, for example, learning that an implausible headline comes from a trusted source should increase the headline’s perceived accuracy. Our observation that providing publisher information increases agreement between laypeople and fact-checkers (albeit by a fairly small amount) supports this theory, because our stimulus set involves mostly implausible headlines. Thus, by this theory, we would expect our experiment to be a situation where publisher information would indeed be helpful, and this is what we observe.

Third, our results contribute to work on applying the wisdom of crowds in political contexts. In particular, we add to the growing body of work suggesting that aggregating judgments can substantially improve performance even in highly politicized contexts. In addition to the studies examining trust in news publishers described above (*20*, *21*), research has shown that crowdsourcing produces more accurate judgments than individual decision-making across a variety of partisan issues including climate change, immigration, and unemployment (*54*, *55*). This result is true whether the network is politically balanced or homogenous, although evidence has shown that a politically balanced group produces higher-quality results than a homogenous one (*54*–*56*). Together, this body of work demonstrates the broad power of crowdsourcing despite systematic polarization in attitudes among members of the crowd. Our results also show the limits of a recent finding that cognitively diverse groups made of a mix of intuitive and analytical thinkers perform better than crowds of only more analytical thinkers (*57*). We do not observe this effect in our data, where mixed groups were no more effective—and if anything, slightly less effective—than groups of only more analytic thinkers.

Last, there are various limitations of our study and important directions for future research. First, we note that our results should not be interpreted as evidence that individual participants identify false information with high reliability. Even when the crowd performance was good, individual participants often misjudged headline veracity.

Second, although we show that small crowds of laypeople perform surprisingly well at identifying (mis)information in our experiment, it is possible that with different design choices layperson crowds could do even better—or that even smaller crowds could achieve similar levels of performance. For example, in the name of scalability, we had the laypeople rate only the headline and lede. It is possible that having them instead read the full article, and/or do research on the claims, could lead to even more accurate ratings. If so, however, the improvement gained would have to be weighed against the increased time required (and thus decreased scalability). Another route to improved performance could be to investigate more complex algorithms for weighting the crowd responses (*31*, *33*, *58*) rather than simply averaging the ratings as we do here. Furthermore, the crowd ratings of headline accuracy could be integrated with other signals, such as publisher quality (*20*, *21*) and existing machine-learning algorithms for misinformation detection.

Third, future work should assess how our results generalize to other contexts. A particular strength of our approach is that the articles we analyzed were selected in an automated fashion by an internal Facebook algorithm, and thus are more representative of articles that platforms need to classify than are the researcher-selected articles used in most previous research. However, we were not able to audit Facebook’s article selection process, and there was little transparency regarding the algorithm used to select the articles. Thus, the articles we analyze here may not actually be representative of misinformation on Facebook or of the content that platforms would use crowds to evaluate. It is possible that biases in the algorithm or the article selection process may have biased our results in some way. For example, certain types of misleading or inaccurate headlines may be underrepresented. Alternatively, it is possible that Facebook misled us and purposefully provided a set of articles that were artificially easy to classify (in an effort to cast their crowdsourcing efforts in a positive light). While we cannot rule out this possibility, we did replicate our results by analyzing a previously published dataset of researcher-selected headlines (*25*) and found that the crowd performed substantially better than for the articles provided by Facebook (see section S16), suggesting that the article set from Facebook was at least more difficult to classify than article sets often used in academic research. That being said, it is critical for future research to apply the approach used here to a wide range of articles to assess the generalizability of our findings. It is possible that under circumstances with rapidly evolving facts, such as in the case of the coronavirus disease 2019 (COVID-19) news environment, results for both the crowd and fact-checkers would differ [although studies with researcher-selected true and false COVID-19 headlines find high crowd performance, e.g., (*39*)].

A related question involves the generalizability of the crowd itself. Our sample was not nationally representative, and it is possible that a representative sample would not perform as well. Similarly, highly inattentive crowds would likely show worse performance. However, our goal here was not to assess the accuracy of judgments of the nation as a whole. Instead, the question was whether it was possible to use laypeople to inexpensively scale fact-checking. In this regard, our results are unambiguously positive. Social media platforms could even simply hire workers from Amazon Mechanical Turk to rate articles, and this would allow for low-cost fact-checking. It remains unclear, however, how these results would generalize to other countries and cultures. Cross-cultural replications are an essential direction for future work. Relatedly, a key feature of the American partisan context that we examine here is that the two relevant factions (Democrats and Republicans) are roughly equally balanced in frequency. As a result, one side’s opinion would not heavily outweigh the other’s when creating average crowd ratings. It is essential for crowdsourcing methods to develop ways to extract signal from the crowd without allowing majorities to certify untruths above marginalized groups (e.g., ethnicity minorities).

Fourth, one might be concerned about partisan crowds purposely altering their responses to try to “game the system” and promote content that they know to be false or misleading but is aligned with their political agenda. However, most Americans do not actually care that much about politics (*59*) and thus are unlikely to be overly motivated to distort their responses. Furthermore, to the extent that partisans do bias their responses, it is likely that they will do so in a symmetric way such that, when creating politically balanced ratings, the bias cancels out. Accordingly, research shows that informing users that their responses will influence what content is shown to social media users does not substantially affect crowd performance for identifying trustworthy news publishers (*21*). However, it is important to keep in mind that the current work focused on settings in which users are presented with specific pieces of content to rate [e.g., Facebook’s “Community Review” (*60*)]. It is unclear how such findings would generalize to designs where participants can choose which pieces of news to rate [e.g., Twitter’s “BirdWatch” (*61*)], which could be more vulnerable to coordinated attacks.

In summary, the experiment presented here indicates the promise of using the wisdom of crowds to scale fact-checking on social media. We believe that, in combination with other measures like detection algorithms, trained experts, and accuracy prompts, crowdsourcing can be a valuable asset in combating the spread of misinformation on social media.

## METHODS

Data and materials are available online (https://osf.io/hts3w/). Participants provided informed consent, and our studies were deemed exempt by the Massachusetts Institute of Technology’s Committee on the Use of Humans as Experimental Subjects, protocol number 1806400195.

### Layperson participants

Between 9 and 11 February 2020, we recruited 1246 U.S. residents from Amazon Mechanical Turk. Of those, 118 participants did not finish the survey and were thus excluded, leaving our final sample with 1128 participants. They had a mean age of 35.18 years old; 38.48% were female; 65.86% had completed at least a bachelor’s degree; 58.33% had an income of less than $50,000; and 54.29% indicated a preference for the Democratic Party over the Republican Party. The median completion time for the full study was 15:48 min, and the median time spent completing the article assessment portion of the study was 7:51 min. Participants were paid a flat fee of $2.25 for their participation. We chose not to give financial rewards for producing answers that agreed with the professional fact-checkers, because one of the benefits of the crowdsourced approach is the ability to avoid claims of liberal bias. If the laypeople were incentivized to agree with the fact-checkers, this would make the crowd susceptible to the same complaints of bias made by some about the fact-checkers.

### Professional fact-checkers

Between 27 October 2019 and 21 January 2020, we hired three professional fact-checkers from the freelancing site Upwork to fact-check our set of articles. These fact-checkers, whom we selected after an extensive vetting process and initial screening task, had substantial expertise and experience, with a combined total of over 2000 hours of fact-checking experience logged on Upwork. We had the fact-checkers first complete an initial assessment task in which they fact-checked 20 of the 207 articles in our set. They were asked to each independently conduct research online to support their evaluations, spending up to 30 min on each article. We then checked their responses to confirm that they were thorough and displayed a mastery of the task, including giving individualized feedback and engaging in discussion when there was substantial disagreement between the fact-checkers. This discussion revealed real, reasoned disagreements, rather than misunderstandings or sloppiness (see section S15 for details). Once this initial trial was completed satisfactorily, we had the fact-checkers independently evaluate the remainder of the articles (without any communication or discussion among themselves about the articles). Furthermore, to demonstrate that our results are not driven by idiosyncrasies of these particular fact-checkers, in section S9, we replicate our main analyses from Fig. 1 using ratings generated by a different set of four professional journalists who had just completed a prestigious fellowship for mid-career journalists and had extensive experience reporting on U.S. politics (*62*). The average rating of these four fact-checkers correlated strongly with the average rating of the Upwork fact-checkers (*r* = 0.81).

### Materials

Participants were each asked to rate the accuracy of 20 articles, drawn at random from a set of 207 articles; professional fact-checkers rated all 207 articles. Our goal is to assess how effective crowdsourcing would be for meeting the misinformation identification challenge faced by social media platforms. Therefore, it is important that the set of articles we use be a good representation of the articles that platforms are trying to classify (rather than, for example, articles that we made up ourselves). To that end, we obtained from Facebook a set of 796 URLs. The articles were sampled from content posted on Facebook in an automated fashion to upsample for content that (i) involved civic topics or health-related information, (ii) was predicted by Facebook’s internal models to be more likely to be false or misleading (using signals such as comments on posts expressing disbelief, false news reports, and pages that have shared misinformation in the past), and/or (iii) was widely shared (i.e., viral). Because we were specifically interested in the effectiveness of layperson assessments based on only reading the headline and lede, we excluded 299 articles because they did not contain a claim of fact in their headline or lede (as determined by four research assistants). We also excluded 34 URLs because they were no longer functional. Of the remaining 463 articles, we randomly selected a subset of 207 to use for our study. The list of URLs can be found in section S1. In terms of URL descriptives, of the 207 URLs, Facebook’s topic classification algorithms labeled 109 as being political, 43 as involving crime and tragedy, 22 as involving social issues, 17 as involving health, and fewer than 15 of all other topic categories (URLs could be classified as involving more than one topic). Furthermore, using MediaBiasFactCheck.org to classify the quality of the source domains for the 209 URLs, 46 were rated Very High or High, 13 were rated Mostly Factual, 75 were rated Mixed, 23 were rated Low, Very Low, or Questionable Source, 12 were rated as Satire, and 38 were not rated by MediaBiasFactCheck.com. Using the quality scores provided by NewsGuard (between 0 and 100), the mean source quality was 67.7 (SD = 28.2), and the median source quality score was 75; 26 URL domains were not rated by NewsGuard.

### Procedure

The layperson participants and the professional fact-checkers completed different, but similar, surveys. In both cases, the survey presented respondents with a series of articles and asked them to assess each article’s central claim. For each article, respondents were first asked to make a categorical classification, choosing between the options “True,” “Misleading,” “False,” and “Not sure.” Second, respondents were asked to provide more fine-grained ratings designed to assess the objective accuracy of the articles using a series of seven-point Likert scales. Specifically, they were asked the extent to which the article (1) described an event that actually happened, (2) was true, (3) was accurate, (4) was reliable, (5) was trustworthy, (6) was objective, and (7) was written in an unbiased way. These seven responses were averaged to construct an aggregate Likert rating (Cronbach’s α = 0.96). Our main text analyses focus on the layperson aggregate Likert ratings as they are much more fine-grained; see section S4 for analyses using the layperson categorical classification ratings, which are qualitatively similar but (as expected) somewhat weaker. Respondents completed the task independently without communication or discussion with each other.

The layperson versus fact-checker surveys differed in how respondents were asked to evaluate the articles. Fact-checkers were presented with the URL of each article and asked to read the full article, and conduct research to evaluate what they assessed to be the article’s central claim. In addition to the ratings described above, the fact-checkers were also asked to provide any relevant evidence from their research that justified their assessment. Laypeople, on the other hand, were only shown the headline and lede sentence of each article, not the full article—and they were not asked to do any research or provide any evidence/sources for their assessments, but rather to rely on their own judgment. Given that mean time per article for laypeople was 35.7 s, median 23.5 s, it is extremely unlikely that many people took it upon themselves to nonetheless research the headlines. Furthermore, to test whether knowledge of the article’s source influenced layperson assessments (*22*), laypeople were randomly assigned to either a No-source condition (just shown headline and lede) or a Source condition (also shown the source domain of the article, e.g., “breitbart.com”).

The layperson versus fact-checker surveys also differed in the number of articles respondents were asked to rate. Each fact-checker rated all 207 articles, while each layperson rated 20 randomly selected articles. On average, each article was rated by 100 laypeople (min, 79; max, 137). After rating the 20 articles, the layperson participants completed the cognitive reflection test (*63*), a political knowledge test, and a series of demographic questions.

### Analysis

A main question of interest for our study is how the average layperson ratings vary based on the number of laypeople included. To assess this question, as well as to achieve politically balanced layperson ratings, we used the following bootstrapping procedure. First, we classified each participant as “Democrat” versus “Republican” based on their response to a question in the demographics about which political party they preferred (six-point scale from “Strong Democrat” to “Strong Republican”; values of 1 to 3 were classified as Democrat, while values of 4 to 6 were classified as Republican, such that no participants were excluded for being Independents).

Then, for each value of *k* layperson ratings per article (from *k* = 2 to *k* = 26), we performed 1000 repetitions of the following procedure. For each article, we randomly sampled (with replacement) *k*/2 Democrats and *k*/2 Republicans. This gave us 1000 different crowds of size *k* for each of the 207 articles. For each crowd, we averaged the responses to create a politically balanced layperson rating for each article. We then computed (i) the correlation across articles between this politically balanced layperson average rating and the average of the fact-checkers’ aggregate Likert ratings, and (ii) an AUC produced by using the politically balanced layperson average ratings for each article to predict whether or not the modal fact-checker categorical rating for that article was “True” (binary variable: 0 = modal response was “False,” “Misleading,” or “Couldn’t be determined”; 1 = “True”). We then report the average value of each of these two outcomes across repetitions, as well as the 95% CI.

In addition, for a crowd of size *k* = 26, we calculated the out-of-sample accuracy of a model that used the politically balanced layperson ratings to predict the fact-checker’s modal binary “Is True” rating (described above). For each of the 1000 different crowds, we performed 20 trials in which we split the data 80/20 into training and test sets, respectively, and calculated the accuracy on the test set of the optimal threshold found in the training set. We then averaged across the 1000 crowds and 20 trials per crowd to calculate the out-of-sample accuracy. A more detailed description of this methodology can be found in sections S2 and S3.

## Supplementary Materials

### This PDF file includes:

Sections S1 to S16 Figs. S1 to S12
