# Supplementary material for: Scaling up fact-checking using the wisdom of crowds
Source: Sci Adv. 2021 Sep 1;7(36):eabf4393. doi: 10.1126/sciadv.abf4393 (PMC8442902; doi:10.1126/sciadv.abf4393)
Supplement: Supplementary file 1 — Sections S1 to S16 Figs. S1 to S12 [file sciadv.abf4393_sm.pdf]

Science Advances
AAAS

Supplementary Materials for

Scaling up fact-checking using the wisdom of crowds

Jennifer Allen, Antonio A. Arechar, Gordon Pennycook, David G. Rand*

*Corresponding author. Email: drand@mit.edu

Published 1 September 2021, *Sci. Adv.* **7**, eabf4393 (2021)
DOI: 10.1126/sciadv.abf4393

This PDF file includes:

Sections S1 to S16
Figs. S1 to S12

1. List of URLs With Fact-checker ratings

URL	Modal Binary Fact-Checker Rating (1 = True, 0 = Not True)	Average Fact-Checker Accuracy Rating (1 - 7 Scale)
http://alexschadenberg.blogspot.com/2018/10/sick-kids-hospital-toronto-will.html	0	3.67
http://coolcatapproves.com/funny/australia-doesnt-exist-and-people-who-live-there-are-actors-paid-by-nasa-flat-earthers-claim/	0	2.14
http://feedytv.com/florida-declares-state-emergency-toxic-red-tide-outbreak.html	1	5.52
http://fortune.com/2016/09/01/medical-marijuana-gun/	1	7.00
http://fox4kc.com/2018/09/28/mom-sues-after-son-doesnt-make-varsity-soccer-team/	1	6.95
http://healthimpactnews.com/2011/dr-russell-blaylock-warns-dont-get-the-flu-shot-it-promotes-alzheimers/	0	1.05
http://nypost.com/2017/09/28/department-of-justice-demands-facebook-account-information-of-anti-trump-activists/	1	4.95
http://nypost.com/2018/09/27/two-men-tell-senate-that-they-not-kavanaugh-assaulted-ford/	1	6.43
http://nypost.com/2018/10/09/de-blasio-signs-bill-allowing-third-gender-on-birth-certificates/	1	7.00
http://pittsburgh.cbslocal.com/2018/10/27/heavy-police-presence-near-synagogue-in-squirrel-hill/	1	7.00
http://rare.us/rare-news/around-the-world/kids-cant-tell-time/	0	4.90
http://rare.us/rare-politics/rare-liberty/police-state/a-court-has-ruled-that-police-can-execute-your-dog-if-it-moves-or-barks/	0	3.62
http://time.com/5390884/nike-sales-go-up-kaepernick-ad/	0	5.33
http://tiphero.com/dont-dress-up-your-chickens/	0	3.67
http://www.55meals.com/did-you-know-your-energy-drinks-contain-bull-urine-semen/	0	1.57
http://www.breakingnews247.net/5b872f00c5639/massive-alligator-found-in-browns-mills-new-jersey.html	0	1.76
http://www.collective-evolution.com/2017/02/13/how-sunscreen-could-be-causing-skin-cancer-not-the-sun/	0	3.71
http://www.higherperspectives.com/one-glass-red-wine-1577145867.html	0	1.95
http://www.icarizona.com/2018/09/arizona-may-end-up-with-former.html	0	3.57
http://www.ladbible.com/community/interesting-a-giant-500ft-asteroid-is-heading-for-earth-at-20000mph-20180826	0	4.67
http://www.ladbible.com/entertainment/music-fans-flip-the-finger-at-machine-gun-kelly-and-get-in-his-face-20180920	1	6.29
http://www.ladbible.com/entertainment/uk-boyfriend-runs-away-after-his-girlfriend-catches-a-brides-bouquet-20180904	1	6.00
http://www.latimes.com/local/lanow/la-me-ln-thousand-oaks-20181107-story.html	1	6.90
http://www.msnbc.com/rachel-maddow-show/trump-crafts-new-excuse-massive-budget-deficit-forest-fires	1	4.57
http://www.pretty52.com/entertaining/tv-and-film-the-one-tree-hill-cast-are-reuniting-for-a-one-hour-special-20180927	1	6.19
http://www.social-consciousness.com/2017/03/donald-trump-says-pedophiles-will-get-death-penalty.html	0	2.43
http://www.theguardian.com/us-news/2018/nov/10/trump-baltics-balkans-mixup-le-monde-belleau-cemetery-paris	1	5.19
http://www.tmz.com/2018/09/18/sesame-street-bert-ernie-gay-couple-confirmed-writer-speculation-over/	1	5.76
http://www.tmz.com/2018/11/01/president-trump-immigrant-caravan-throwing-rocks-same-firearms/	0	2.29
http://www.wdbj7.com/content/news/Farmers-Almanac-predicts-a--491821281.html	1	5.29
http://www.wect.com/2018/10/17/breaking-state-trooper-shot-killed-suspect-custody/	1	6.90

http://www.worldstarhiphop.com/videos/video.php?v=wshhddDiUTw9SDG7wvd7	0	1.57
https://abc13.com/4038817/	1	6.90
https://abc13.com/4554120/	1	6.95
https://abcnews.go.com/Politics/blame-abc-news-finds-17-cases-invoking-trump/story?id=58912889	1	3.67
https://abcnews.go.com/Politics/desantis-floridians-monkey-electing-african-american-democrat-governor/story?id=57476957	0	6.43
https://abcnews.go.com/Politics/trump-kicks-off-week-tweet-calling-media-true/story?id=58827743	1	6.43
https://abcnews.go.com/Politics/trump-plans-end-birthright-citizenship-babies-born-citizens/story?id=58845684	1	6.76
https://abcnews.go.com/US/daycare-owner-drugged-kids-tied-car-seats-police/story?id=57932436	1	6.81
https://abcnews.go.com/US/man-accused-groping-woman-flight-trump-grab-women/story?id=58681265	1	6.95
https://americanmilitarynews.com/2018/08/china-hacked-hillary-clintons-email-server-and-took-nearly-all-her-emails-report-says/	0	3.62
https://americanmilitarynews.com/2018/10/armys-nco-promotion-criteria-fails-to-gauge-leadership-qualities-study-says/	1	7.00
https://americanmilitarynews.com/2018/10/disabled-and-retired-vets-to-see-largest-cost-of-living-raise-in-six-years/	1	6.67
https://americanmilitarynews.com/2018/10/guatemala-captured-100-isis-terrorists-president-reveals-ahead-of-migrant-caravan-arrival/	0	4.48
https://babylonbee.com/news/bill-clinton-allegations-of-sexual-misconduct-should-disqualify-a-man-from-public-office	0	1.86
https://babylonbee.com/news/joel-osteen-launches-line-pastoral-wear-sheeps-clothing/	0	1.86
https://babylonbee.com/news/socialist-leaders-clarify-we-only-want-socialism-for-everyone-else/	0	1.52
https://conservativedailypost.com/savage-claims-ford-deeply-tied-to-deep-state/	0	3.00
https://conventionofstates.com/news/ginsburg-can-t-remember-14th-amendment-gets-pocket-constitution-from-the-audience	0	3.81
https://dailycaller.com/2018/10/15/elizabeth-warren-less-native-american-dna/	0	4.10
https://dailycaller.com/2018/10/30/bus-mexico-migrant-caravan-border/	0	3.95
https://educateinspirechange.org/health/experienced-butcher-admits-see-cancer-pork-just-cut-still-sell-customers/	1	5.43
https://endoftheageheadlines.wordpress.com/2018/10/24/deep-state-sending-explosive-packages-to-themselves-in-hopes-of-stopping-red-wave/	0	2.24
https://kutv.com/news/local/woman-who-alleges-mtc-president-raped-her-filmed-testifying-about-rape-in-church	1	6.67
https://lawandcrime.com/civil-rights/gop-removes-sole-polling-place-from-famous-hispanic-majority-city-in-kansas/	1	5.05
https://legalinsurrection.com/2018/09/maxine-waters-suggests-knocking-off-trump-then-going-after-pence/	0	4.14
https://meaww.com/queen-invites-meghan-markle-mother-doria-christmas-sandringham	0	4.67
https://neonnettle.com/news/4335-obama-at-bilderberg-the-us-must-surrender-to-the-new-world-order-	0	1.00
https://news.jamaicans.com/kanye-west-bob-marleys-spirit-flows/	1	6.29
https://news.unclesamsmisguidedchildren.com/obama-says-benghazi-is-a-wild-conspiracy-theory/	0	2.90
https://news.vice.com/en_us/article/3kek75/another-kavanaugh-accuser-is-taking-to-maryland-authorities	1	6.52
https://news.vice.com/en_us/article/nepwng/some-texans-had-to-wait-so-long-to-vote-they-gave-up-a-lawsuit-is-trying-to-give-them-a-second-chance	1	6.76
https://patriotjournal.org/democrat-fbi-closet/	0	2.67
https://patriotjournal.org/sanctuary-state-vote-trump/	0	2.52
https://patriotjournal.org/video-train-south-border/	0	3.86
https://prepforthat.com/christine-blasey-fords-yearbook-seems-to-show-high-school-racism/	0	3.24
https://realfarmacy.com/surgeon-mammogram/	0	4.19
https://rewire.news/ablc/2018/10/11/supreme-court-native-americans-november/	1	5.38

https://rwnofficial.com/dems-huge-secret-hillary-caught-in-special-relationship-with-stormy-daniels/	0	2.43
https://shareblue.com/russia-president-vladimir-putin-annual-address-americas-downfall/	0	2.67
https://socialsecurityworks.org/2018/04/12/politicians-steal-social-security/	0	2.24
https://thefederalistpapers.org/opinion/california-bans-feeding-homeless-without-police-supervision	1	4.33
https://thefederalistpapers.org/us/liberal-professor-says-hurricane-victims-deserved-got-supporting-republicans	0	3.86
https://thefreethoughtproject.com/watch-cop-chokes-innocent-man-calling-water-company-corruption/	0	2.57
https://tribunist.com/news/band-performs-skit-about-shooting-police-during-halftime-of-football-game/	0	3.10
https://twitter.com/realDonaldTrump/status/1040217897703026689	0	3.67
https://washingtonpress.com/2018/10/28/pittsburgh-jewish-leaders-just-banned-trump-from-their-city-until-he-meets-their-demands/	0	3.05
https://wokesloth.com/fox-news-poll/stefanie/	0	1.71
https://worldnewsdailyreport.com/canadians-face-major-donut-shortage-after-first-day-of-cannabis-legalization/	0	1.76
https://worldnewsdailyreport.com/cops-beat-up-teen-after-bank-teller-mistakes-his-erection-for-a-pistol/	0	1.86
https://worldnewsdailyreport.com/man-accused-of-raping-a-cow-claims-it-is-the-reincarnation-of-his-dead-wife/	0	1.57
https://worldnewsdailyreport.com/morgue-worker-arrested-after-giving-birth-to-a-dead-mans-baby/	0	1.86
https://worldnewsdailyreport.com/pregnant-teen-seeks-13-paternity-tests-after-gangbang-with-football-team/	0	1.86
https://worldnewsdailyreport.com/teen-on-female-viagra-crashes-into-building-while-masturbating-to-gear-shift/	0	1.76
https://worldnewsdailyreport.com/teenager-sues-his-parents-for-250000-for-naming-him-gaylord/	0	2.05
https://worldnewsdailyreport.com/woman-arrested-for-training-squirrels-to-attack-her-ex-boyfriend/	0	1.95
https://worldnewsdailyreport.com/woman-claims-she-is-the-daughter-of-marilyn-monroe-and-jfk/	0	1.71
https://www.12up.com/posts/6209256-report-steelers-hoping-eagles-renew-their-interest-in-potential-trade-for-le-veon-bell	1	6.33
https://www.aol.com/article/news/2015/12/09/study-links-e-cigarettes-to-incurable-disease-called-popcorn-lu/21281029/	1	6.81
https://www.axios.com/rod-rosenstein-resign-justice-department-trump-cf761f4c-fca3-4794-92d4-a56c9e32ff43.html	1	5.38
https://www.bet.com/celebrities/news/2016/12/07/kim-kardashian-kanye-west-marriage.html?cid=facebook	0	2.76
https://www.billboard.com/articles/columns/chart-beat/8094819/cardi-b-beyonce-five-hits-top-10-rb-hip-hop-songs-chart	1	6.00
https://www.breitbart.com/big-journalism/2018/08/18/cnn-accused-intimidating-paul-manafort-jury/	0	3.14
https://www.breitbart.com/border/2018/10/30/armed-migrants-in-caravan-opened-fire-on-mexican-cops-say-authorities/	0	4.19
https://www.breitbart.com/entertainment/2018/10/22/bette-midler-world-under-siege-from-murderers-like-trump/	1	4.81
https://www.breitbart.com/midterm-election/2018/10/18/nancy-pelosi-collateral-damage/	0	2.48
https://www.breitbart.com/politics/2018/10/14/cnn-poll-biden-leads-field-2020-democratic-hopefuls/	1	5.10
https://www.breitbart.com/politics/2018/11/01/orourke-campaign-exposed-in-undercover-video-for-assisting-honduran-migrants-nobody-needs-to-know/	0	4.00
https://www.breitbart.com/the-media/2018/10/26/nolte-nbc-news-hid-info-wouldve-cleared-kavanaugh-avenatti-rape-allegations/	1	4.10
https://www.breitbart.com/video/2018/10/18/biden-there-is-no-evidence-of-widespread-voter-fraud-in-the-american-electoral-process/	1	7.00

https://www.breitbart.com/video/2018/10/22/obama-unlike-some-i-state-facts-i-dont-believe-in-just-making-stuff-up/	1	6.81
https://www.buzzfeed.com/aliciabarron/this-reeses-machine-will-swap-out-your-halloween-candy-for	1	6.81
https://www.cbsnews.com/video/forecast-tropical-storm-erika-could-hit-florida-as-hurricane/	1	6.33
https://www.chicksonright.com/youngconservatives/2018/10/16/democrats-eat-crow-fbi-raids-city-offices-headed-by-san-juan-mayor-trump-was-right-all-along/	0	4.43
https://www.chicksonright.com/youngconservatives/2018/10/24/time-to-be-polite-is-over-heated-fox-co-hosts-confront-juan-williams-on-air-if-youre-going-to-sit-there-and-call-your-colleague-a-liar/	0	3.52
https://www.christianpost.com/news/china-trying-to-rewrite-the-bible-force-churches-sing-communist-anthems-227664/	1	6.43
https://www.christianpost.com/news/white-house-hosts-100-evangelical-leaders-state-like-dinner-this-is-spiritual-warfare-227044/	1	6.43
https://www.cnbc.com/2018/10/19/saudi-arabia-admits-journalist-jamal-khashoggi-was-killed-after-a-fight-broke-out-in-consulate.html	1	6.86
https://www.cnn.com/2018/10/16/us/first-female-commander-us-army-trnd/index.html	1	6.86
https://www.cnn.com/2018/10/24/us/florida-middle-girls-allegedly-wanted-to-kill-classmates/index.html	1	6.81
https://www.concealedcarry.com/news/armed-citizens-are-successful-95-of-the-time-at-active-shooter-events-fbi/	0	3.57
https://www.dailymail.co.uk/health/article-6176151/No-evidence-having-high-levels-bad-cholesterol-causes-heart-disease.html	0	4.62
https://www.dailymail.co.uk/news/article-6285989/Canada-kicks-muted-pot-party-1st-G7-nation-OK-recreational-cannabis.html	1	6.57
https://www.dailymail.co.uk/news/article-6373429/Caitlyn-Jenners-Malibu-house-burns-California-wildfires-rage-control.html	1	6.52
https://www.dailywire.com/news/34778/medical-website-indulges-trans-community-new-term-hank-berrien	0	3.90
https://www.dailywire.com/news/34945/blowin-wind-hospital-security-guard-fired-hank-berrien	1	6.76
https://www.dailywire.com/news/35040/controversial-reporter-jemele-hill-out-espn-part-emily-zanotti	1	5.86
https://www.dailywire.com/news/35097/la-and-ny-overrun-topless-women-seeking-equality-amanda-prestigiacomo	1	5.10
https://www.dailywire.com/news/35631/linda-sarsour-calls-dehumanization-jews-report-ryan-saavedra	0	3.67
https://www.dailywire.com/news/36198/watch-beto-orourke-pins-himself-corner-over-drunk-ryan-saavedra	1	6.48
https://www.dailywire.com/news/37070/anti-kavanaugh-protester-uses-her-two-kids-she-hank-berrien	1	4.62
https://www.dailywire.com/news/37398/migrant-caravan-swells-5000-resumes-advance-us-joseph-curl	1	5.19
https://www.dailywire.com/news/37552/migrant-caravan-marching-us-borders-swells-14000-joseph-curl	0	4.86
https://www.dailywire.com/news/37685/epa-greenhouse-gas-emissions-dropped-nearly-3-joseph-curl	0	4.86
https://www.dailywire.com/news/37900/nobody-needs-know-orourke-campaign-appears-ryan-saavedra	0	4.33
https://www.dailywire.com/news/38153/breaking-voter-fraud-allegedly-found-deep-blue-ryan-saavedra	0	4.19
https://www.dailywire.com/news/38230/broward-county-elections-supervisor-be-forced-james-barrett	1	5.00
https://www.forbes.com/sites/rachellebergstein/2016/11/10/new-balance-gets-political-and-throws-support-behind-trump/	1	6.52
https://www.foxnews.com/politics/mob-chants-threats-outside-tucker-carlsons-dc-home	1	6.52
https://www.hannity.com/media-room/free-money-cory-booker-unveils-plan-to-give-poor-americans-50000/	0	4.43
https://www.hatetriots.com/2018/10/nancy-pelosi-shouted-out-of-restaurant.html	0	3.33

https://www.healthnutnews.com/60-lab-studies-now-confirm-cancer-link-to-a-vaccine-you-probably-had-as-a-child/	0	2.00
https://www.healthy-holistic-living.com/instant-noodles-inflammation-dementia.html	0	2.52
https://www.iflscience.com/editors-blog/pieces-of-a-ufo-fell-from-the-sky-and-landed-in-remote-cambodian-village/	1	5.81
https://www.iflscience.com/health-and-medicine/a-cancer-kill-switch-has-been-found-in-the-body-and-researchers-are-already-hard-at-work-to-harness-it/	1	7.00
https://www.iflscience.com/plants-and-animals/drone-footage-reveals-over-100-whales-trapped-in-secret-underwater-jails/	1	6.90
https://www.investors.com/politics/editorials/u-s-has-3-5-million-more-registered-voters-than-live-adults-a-red-flag-for-electoral-fraud/	0	4.10
https://www.joyscribe.com/all-of-the-harry-potter-films-are-officially-coming-to-netflix/	0	5.19
https://www.jta.org/2018/10/27/top-headlines/trump-blames-deaths-pittsburgh-synagogue-lack-armed-guards	1	6.81
https://www.judicialwatch.org/press-room/press-releases/judicial-watch-uncovers-more-classified-material-on-hillary-clintons-unsecure-email-system/	1	5.67
https://www.lapd.com/blog/lapd-officer-shot-point-blank-range-and-not-peep-aclu-blm-or-assemblymember-weber	1	3.90
https://www.lifenews.com/2018/08/20/oprah-winfrey-promotes-shout-your-abortion-movement-where-women-brag-about-their-abortions/	0	3.19
https://www.lifenews.com/2018/08/27/125-women-take-abortion-pills-to-kill-their-babies-to-protest-pro-life-laws/	0	2.48
https://www.lifenews.com/2018/08/28/united-methodist-church-proposes-new-position-statement-saying-we-support-abortion/	0	3.86
https://www.lifenews.com/2018/09/14/chelsea-clinton-says-it-would-be-un-christian-to-protect-babies-from-abortion/	0	3.43
https://www.lifenews.com/2018/09/17/joe-biden-calls-trump-supporters-the-dregs-of-society/	0	3.57
https://www.lifezette.com/2018/10/kavanaugh-turned-down-scads-of-gofundme-dollars-blasey-ford-hits-paydirt/	0	4.14
https://www.lifezette.com/2018/11/trump-challenges-dems-on-kavanaugh-accusers-admission-that-she-made-it-all-up-where-are-you-on-this/	0	4.10
https://www.lifezette.com/2018/11/winner-ocasio-cortez-insists-of-medicare-for-all-you-just-pay-for-it/	1	4.05
https://www.lovebscott.com/steve-harveys-talk-show-canceled-nbc-will-replaced-kelly-clarkson-show	1	6.90
https://www.metrotimes.com/table-and-bar/archives/2018/10/18/detroit-judge-tosses-gardening-while-black-case-brought-by-three-white-women	1	6.43
https://www.movieguide.org/news-articles/netflix-animated-series-dedicates-an-entire-episode-to-promote-planned-parenthood-and-killing-babies.html	0	3.38
https://www.msn.com/en-us/news/us/jewish-leaders-tell-trump-hes-not-welcome-in-pittsburgh-until-he-denounces-white-nationalism/ar-BBP2lEL	1	6.05
https://www.myleaderpaper.com/news/accidents/desloge-man-dies-in-two-vehicle-accident-west-of-festus/article_f2b6b228-dbdd-11e8-8b87-abb2d7153695.html	1	7.00
https://www.nbcnews.com/news/us-news/meijer-pharmacist-denies-michigan-woman-miscarriage-medication-citing-religious-beliefs-n921711	1	7.00
https://www.nbcnews.com/news/us-news/retired-firefighter-who-fired-shotgun-black-teen-asking-directions-gets-n935611	1	6.90
https://www.nbcnews.com/politics/donald-trump/least-impressive-sex-i-ever-had-stormy-daniels-tells-all-n910566	1	6.10
https://www.nbcnews.com/pop-culture/celebrity/burt-reynolds-charismatic-star-1970s-blockbusters-dies-82-n907206	1	7.00
https://www.nbcnews.com/tech/social-media/far-right-group-takes-victory-lap-social-media-after-violence-n920506	1	5.05
https://www.nbcnews.com/video/cincinnati-police-tased-11-year-old-girl-accused-of-shoplifting-1313262659520?v=raila&	1	6.95
https://www.nbcnews.com/video/sessions-says-he-will-recuse-himself-from-any-clinton-investigations-851626564002?v=railb	1	7.00
https://www.newsandguts.com/white-house-leaves-pence-condemn-attacks-clinton-obama-cnn/	0	2.38

https://www.newsandguts.com/white-house-uses-doctored-video-support-claim-reporter/	1	5.86
https://www.newsweek.com/donald-trump-jr-says-joe-biden-went-too-far-calling-trump-voters-dregs-1123294	1	6.05
https://www.newsweek.com/donald-trump-speak-anti-lgbt-hate-groups-annual-event-first-president-683927	1	3.52
https://www.newsweek.com/trump-ceo-pay-wages-tax-cuts-1076795	1	5.86
https://www.newsweek.com/witches-hex-brett-kavanaugh-amy-kremer-scary-conservatives-ritual-trump-1168948	0	2.81
https://www.newyorker.com/news/news-desk/the-five-year-old-who-was-detained-at-the-border-and-convinced-to-sign-away-her-rights	1	5.90
https://www.nsfnews.com/5b8ea8e312074/jackson-man-arrested-for-hacking-a-college-computer-and-returning-all-funds-to-students-since-2010.html	0	2.05
https://www.nytimes.com/2018/05/22/us/politics/georgia-primary-abrams-results.html	1	6.24
https://www.pbs.org/newshour/politics/trump-says-it-will-be-hard-to-unify-country-without-a-major-event	1	6.95
https://www.pbs.org/newshour/politics/watch-kanye-west-in-white-house-visit-says-maga-hat-gives-him-power	1	5.62
https://www.plantbasednews.org/post/no-one-should-be-doing-keto-diet-leading-cardiologist	0	4.62
https://www.politico.com/states/florida/story/2017/12/15/experts-browards-elections-chief-broke-law-in-destroying-ballots-150258	1	6.19
https://www.politicususa.com/2018/10/26/texas-voting-machines-beto-orourke-ted-cruz.html	0	3.29
https://www.rawstory.com/2018/11/woman-pretends-persecuted-trump-supporter-scams-conservatives-thousands-dollars/	0	3.71
https://www.rd.com/food/fun/coffee-black-psychopath-study/	1	6.24
https://www.rollingstone.com/politics/politics-news/brian-kemp-leaked-audio-georgia-voting-745711/	1	6.00
https://www.scarymommy.com/parkland-shooting-victim-sculpture/	1	6.05
https://www.thedailybeast.com/elizabeth-warren-releases-native-american-dna-test-results	1	5.62
https://www.thedailybeast.com/ivanka-trumps-gurus-say-their-techniques-can-end-war-and-make-you-fly	0	5.71
https://www.thedenverchannel.com/news/chris-watts-case/chris-watts-reaches-plea-deal-to-avoid-death-penalty-in-deaths-of-pregnant-wife-2-daughters	1	6.90
https://www.theepochtimes.com/joy-disbelief-as-korean-families-separated-by-war-meet-after-65-years_2628430.html	1	6.95
https://www.theepochtimes.com/threat-of-yellowstone-volcano-eruption-increases-to-high-says-usgs_2700008.html	1	6.38
https://www.theepochtimes.com/top-ranking-democrats-silent-on-keith-ellison-abuse-claims_2669521.html	0	4.33
https://www.thegatewaypundit.com/2018/09/former-scalia-law-clerk-drops-pictures-and-evidence-that-blows-christine-fords-case-wide-open/	0	2.62
https://www.thehollywoodgossip.com/2018/07/meghan-markle-and-kate-middleton-due-to-give-birth-on-the-same-d/	0	3.90
https://www.thenewamerican.com/usnews/crime/item/24549-nypd-source-weiner-laptop-has-enough-evidence-to-put-hillary-away-for-life	0	2.48
https://www.theroot.com/trump-rants-like-racist-grandpa-in-speech-to-maga-negro-1830045693	0	2.71
https://www.thisisinsider.com/hot-cocoa-rolls-pillsbury-2018-11	1	5.95
https://www.thisisinsider.com/michael-buble-quits-music-sons-cancer-battle-2018-10	0	4.48
https://www.unilad.co.uk/tv/bert-and-ernie-are-a-gay-couple-confirms-sesame-street-writer/	0	5.33
https://www.unilad.co.uk/tv/making-a-murderer-part-2-to-be-officially-released-on-october-19th-2018/	1	6.57
https://www.usatoday.com/story/news/nation-now/2018/08/18/no-prison-time-ex-houston-doctor-who-raped-heavily-sedated-patient/1031665002/	1	6.57
https://www.usatoday.com/story/news/politics/2018/08/30/federal-pay-freeze-trump-cancels-2-1-percent-pay-raise-federal-workers-citing-budget-deficit/1145355002/	1	6.86
https://www.vice.com/en_us/article/43ejmj/generation-z-is-skipping-college-for-trade-school	1	5.33

https://www.washingtonpost.com/technology/2018/11/08/white-house-shares-doctored-video-support-punishment-journalist-jim-acosta/	1	6.57
https://www.washingtontimes.com/news/2018/oct/14/ted-wheeler-portland-mayor-stands-decision-allow-a/	1	5.19
https://www.westernjournal.com/ct/cnn-fails-blunder-kavanaugh/	1	4.81
https://www.westernjournal.com/ct/huge-feinstein-investigated/	0	3.67
https://www.westernjournal.com/ct/trump-black-liberal-reporter-racist-question/	1	3.57
https://www.westernjournal.com/ct/watch-slow-mo-video-catches-angry-acosta-shoving-woman-white-house/	0	1.76
https://www.westernjournal.com/obama-urges-lives-better-non-americans/	1	6.00
https://www.yahoo.com/entertainment/orange-new-black-cancelled-netflix-085607387.html	1	6.52
https://www.yahoo.com/lifestyle/low-carb-diet-keto-could-134200344.html	1	6.29
https://www.yahoo.com/news/trump-helped-parents-hide-money-tax-returns-york-195327106.html	1	6.62
https://www.zeptha.com/cotton-swab-soaked-in-alcohol-and-placed-in-your-navel/	0	1.62

2. Methods: Bootstrapping Many Crowds

In order to simulate the performance of crowds of different sizes, we performed the bootstrapping procedure described below. Note that we sampled the layperson judgments independently for each article, rather than keeping the same crowd for the entire set of 207 articles. We did this because we collected only 20 ratings per layperson, similar to the implementation likely to be used by platforms in which laypeople would rate only a subset of all content. Simulations were performed in R using the purrr, foreach, doParallel packages, and code can be found on our OSF site: https://osf.io/hts3w/.

K = the maximum size of the crowd (K = 26)
N = the total number of articles in the set ($N = 207$)
B = the total number of bootstraps for each article ($B = 1000$)

R_i = The set of Democratic layperson judgments for article i
D_i = The set of Republican layperson judgments for article i

f_i = The average continuous fact-checker rating for article i
m_i = The average binary truth fact-checker rating for article i (0 = Not True, 1 = True)

For $k = 2 \ldots K$:
    For $b = 1 \ldots B$:
        For i = $1 \ldots N$:
            $R_{i,b,k}$ = Sample with replacement k / 2 responses from R_i
            $D_{i,b,k}$ = Sample with replacement k / 2 responses from D_i
            $\mu_{i,b,k}$ = Average of $\{R_{i,k,b}, D_{i,k,b}\}$
        $\rho_{k,b}$ = Pearson Correlation between the average layperson ratings $\{\mu_{i,b,1} \ldots \mu_{i,b,n}\}$ and the fact-checker ratings $\{f_1 \ldots f_n\}$ and
        $a_{k,b}$ = AUC of a model using the average layperson ratings $\{\mu_{i,b,1} \ldots \mu_{i,b,n}\}$ to predict the categorical fact-checker ratings $\{m_1 \ldots m_n\}$
    r_k = Average Pearson Correlation across all bootstraps $\{\rho_{k,1} \ldots \rho_{k,B}\}$
    AUC_k = Average AUC across all bootstraps $\{a_{k,1} \ldots a_{k,B}\}$

3. Methods: Out-of-Sample Accuracy

In order to estimate the out-of-sample accuracy of a model that uses the crowd's aggregate ratings to predict the fact-checkers' average binary rating as a function of the percent of unanimous headlines in the dataset, we performed the procedure described below. In order to calculate the out-of-sample accuracy, we first calculated the average layperson ratings for a crowd of size 26 for each article, averaged across 1000 bootstrapped simulations. We then split our headlines data into an 80/20 train/test set, calculated the optimal cutoff that maximized the weighted average of accuracy on unanimous and non-unanimous headlines, and then used that cutoff to calculate the weighted out-of-sample accuracy. The following procedure was done in Python using the pandas and numpy packages. Code can be found on our OSF site: https://osf.io/hts3w/.

S = Number of test/train split trials
C = The set of possible cutoffs, taken from the average fact-checker rating of each of the 207 articles
p = The proportion of unanimous headlines in the sample
max_c_p = Optimal cutoff based on training data where there are p unanimous articles in the sample
L = The set of laypeople ratings where l_j is the rating for headline j
F = The set of fact-checker ratings where f_j is the rating for headline j
U = The set of articles with unanimous fact-checker agreement
NU = The set of articles with non-unanimous fact-checker agreement

For i in 1..S:
  Split headlines into 80/20 train/test sets where:
  $U_{i,train}$ = Set of unanimous headlines for training
  $NU_{i,train}$ = Set of non-unanimous headlines for training
  $U_{i,test}$ = Set of unanimous headlines for testing
  $NU_{i,test}$ = Set of non-unanimous headlines for testing
  For p in 0...1 by .01:
    For c in C:

$$Acc_{train,c,p,i} = p * \frac{1}{|Utrain|}\sum_{j \epsilon Utrain} \mathbf{1}((l_j > c) == f_j) + (1 - p) * \frac{1}{|NUtrain|}\sum_{j \,\epsilon\, NUtrain} \mathbf{1}((l_j > c) == f_j)$$

$$max\_c_p = \text{argmax}_c\, Acc_{train,c,p,i}$$

$$Acc_{test,p,i} = p * \frac{1}{|Utest|}\sum_{j \epsilon Utest} \mathbf{1}((l_j > max\_c_p) == f_j) + (1 - p) * \frac{1}{|NUtest|}\sum_{j \,\epsilon\, NUtest} \mathbf{1}((l_j > max\_c_p) == f_j)$$

$$Acc_{test,p} = \text{Average}\ \{Acc_{test,p,1} \ldots Acc_{test,p,S}\}$$

4. Robustness: Predicting fact-Checker binary ratings with laypeople binary ratings

As a robustness test, we performed the same bootstrapping procedure as specified in Section S2 to calculate the AUC of a model that uses the categorical ratings of the laypeople, rather than the average continuous ratings, to predict the categorical ratings of the fact-checkers. We binarize the crowd's ratings in the same way as the fact-checkers with 1 as True and 0 as Not True and use the proportion of True ratings in the crowd to predict the fact-checkers ratings.

As one might expect, the results are similar to - but slightly worse than - the model that uses the (more sensitive) continuous crowd ratings. The AUC for the model on the unanimous set asymptotes at .90 for the source condition, and .85 for the non-source condition, slightly lower than the .92 and .90 from the continuous model, respectively. The performance on the non-unanimous set shows a similar pattern, .74 for the source condition and .71 for no source vs. .78 and .74 for the continuous model, respectively.

Unsurprisingly, the model is worse for smaller crowds than the continuous model, showing an average AUC that is 0.05 - 0.1 points worse than the continuous model for a crowd of size 2. However, with larger crowds, the model does better, narrowing the gap to about .02 - .05. Overall, the results still show that even a binary rating can be a useful predictor when aggregated across many people.

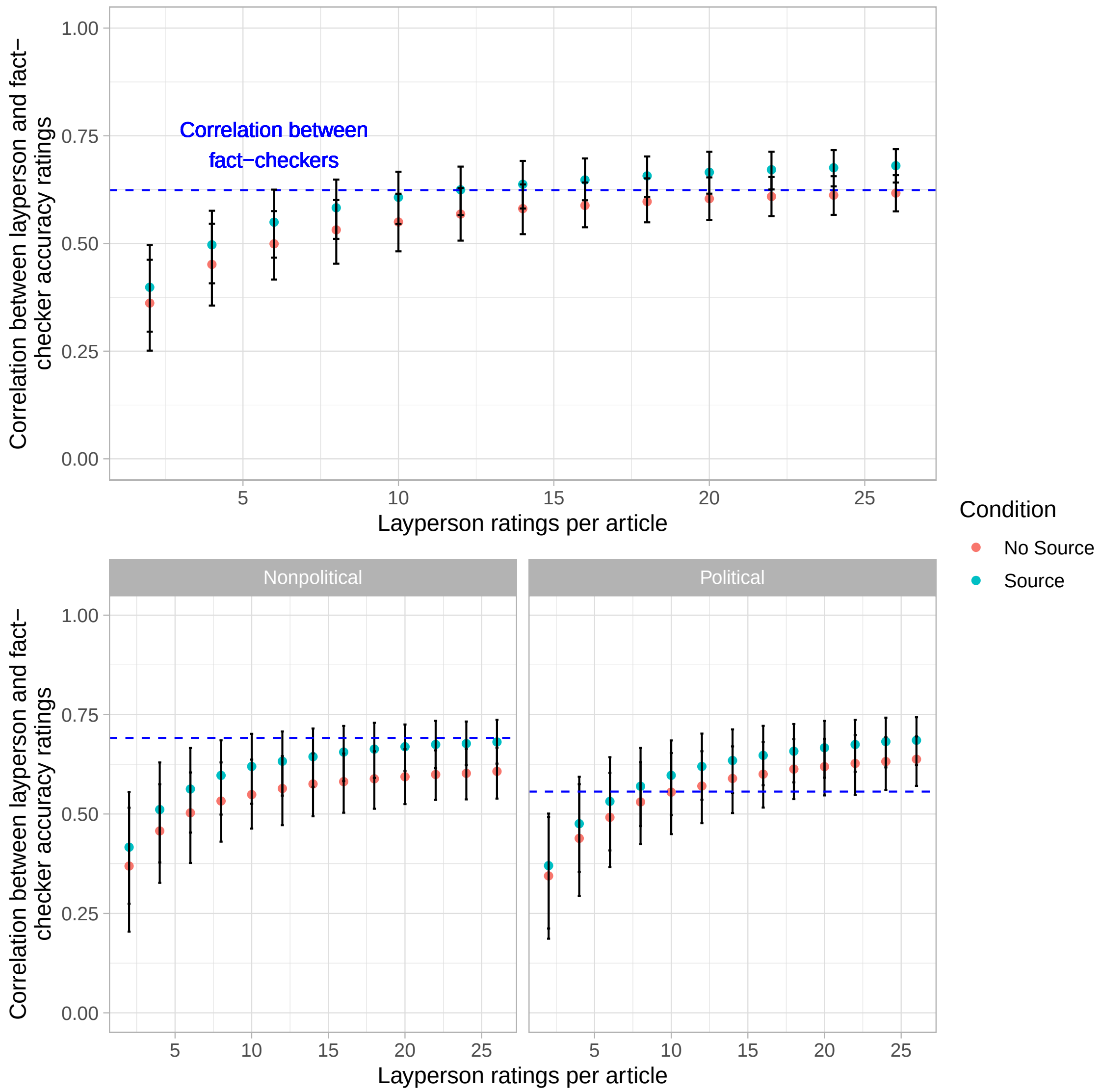

Figure S1. *Classifying articles as true versus non-true based on layperson aggregate categorical ratings. (a) (b) AUC scores as a function of the number of layperson ratings per article and source condition. AUC is calculated using a model in which the layperson rating is coded as 1 for True and 0 Otherwise and the share of "True" layperson ratings is used to predict the modal fact-checker categorical rating, coded in the same way.*

5. Robustness: Predicting Fact-checker "Is False" ratings

While predicting whether a URL is "True" vs. "False" or "Misleading" is a more relevant task for platforms combating misinformation, there are also cases when distinguishing outright "False" from "Misleading" or "True" URLs is useful. Thus, we extend the bootstrapping procedure described in Section S1 to use the crowd's average Likert-scale accuracy ratings to predict the fact-checkers' categorical ratings where "False" is coded as 1 and all other options are coded as 0.

The results are shown in Figure S2. The results are qualitatively similar to the same model predicting "Is True". However, the performance is slightly worse, with the AUC asymptoting at .85 - .86 if or unanimous URLs and .74 - .75 for non-unanimous URLs, as compared to .90 - .92 for unanimous and .74 - .78 for non-unanimous in the "Is True" prediction case. The results from the source condition do not significantly differ from the no-source condition.

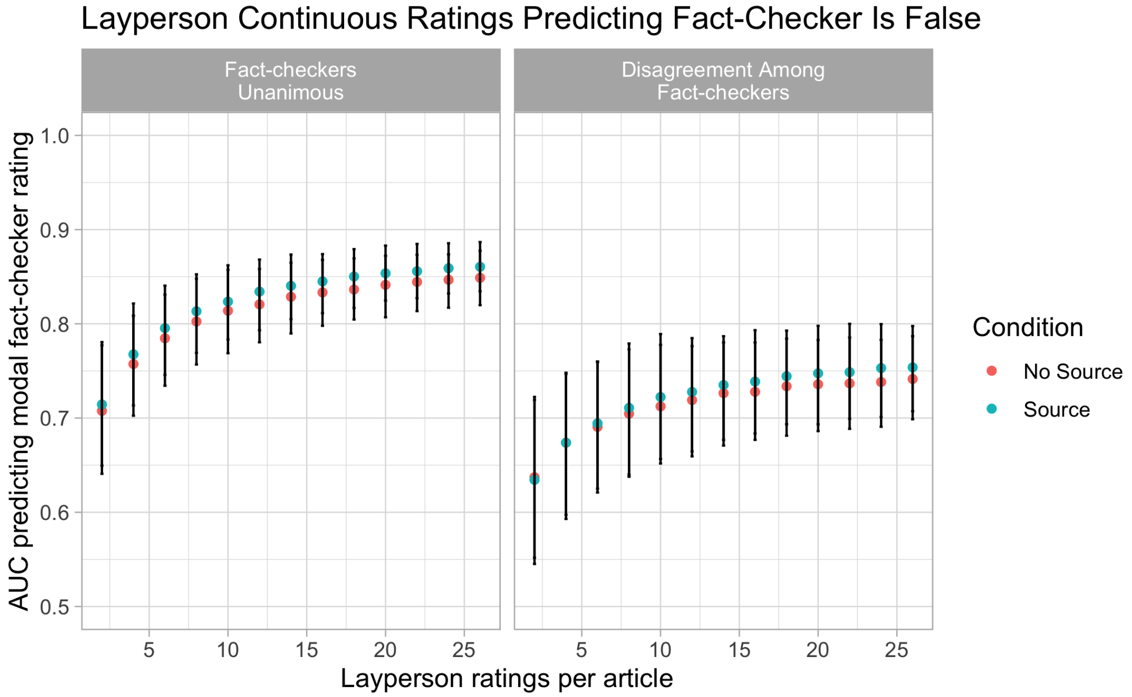

Figure S2. *Classifying articles as false versus non-false based on layperson aggregate continuous ratings. (a) (b) AUC scores as a function of the number of layperson ratings per article and source condition. AUC is calculated using a model in which the layperson average Likert-scale accuracy ratings are used to predict the modal fact-checker categorical rating, with responses coded as 1 for False and 0 Otherwise.*

6. Robustness: AUC, Political vs. Non-Political Headlines

We also examine the AUC of a model predicting the modal fact-checker rating, with "True" coded as 1 and 0 otherwise. We do not see a significant difference between the performance of the crowd on political vs. non-political headlines in either condition.

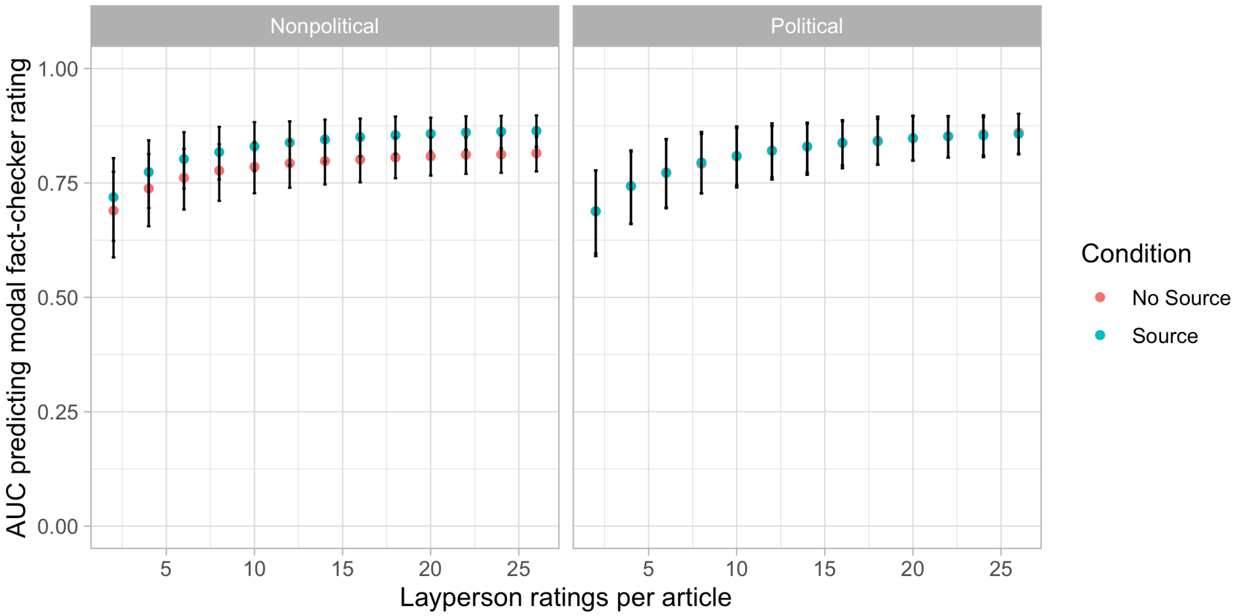

Figure S3. *Classifying articles as true versus non-true based on layperson aggregate continuous ratings. (a) (b) AUC scores as a function of the number of layperson ratings per article and source condition. Panels show results for (a) Non-Political articles, (B) Political Articles.*

7. Robustness: Political Knowledge and Cognitive Reflection by Party

We also examine whether the results that show that crowds with high levels of political knowledge and cognitive reflection outperform their low level counterparts hold among both Democrats and Republicans. Figure S3 shows that high CRT and high political knowledge crowds outperform their low level counterparts for balanced, all Democrat, and all Republican crowds. Note that we look at the performance of a crowd of size 10, rather than size 26 as in Figure 3 the main text, because restricting participants to be of only one party cuts the pool of potential respondents in half.

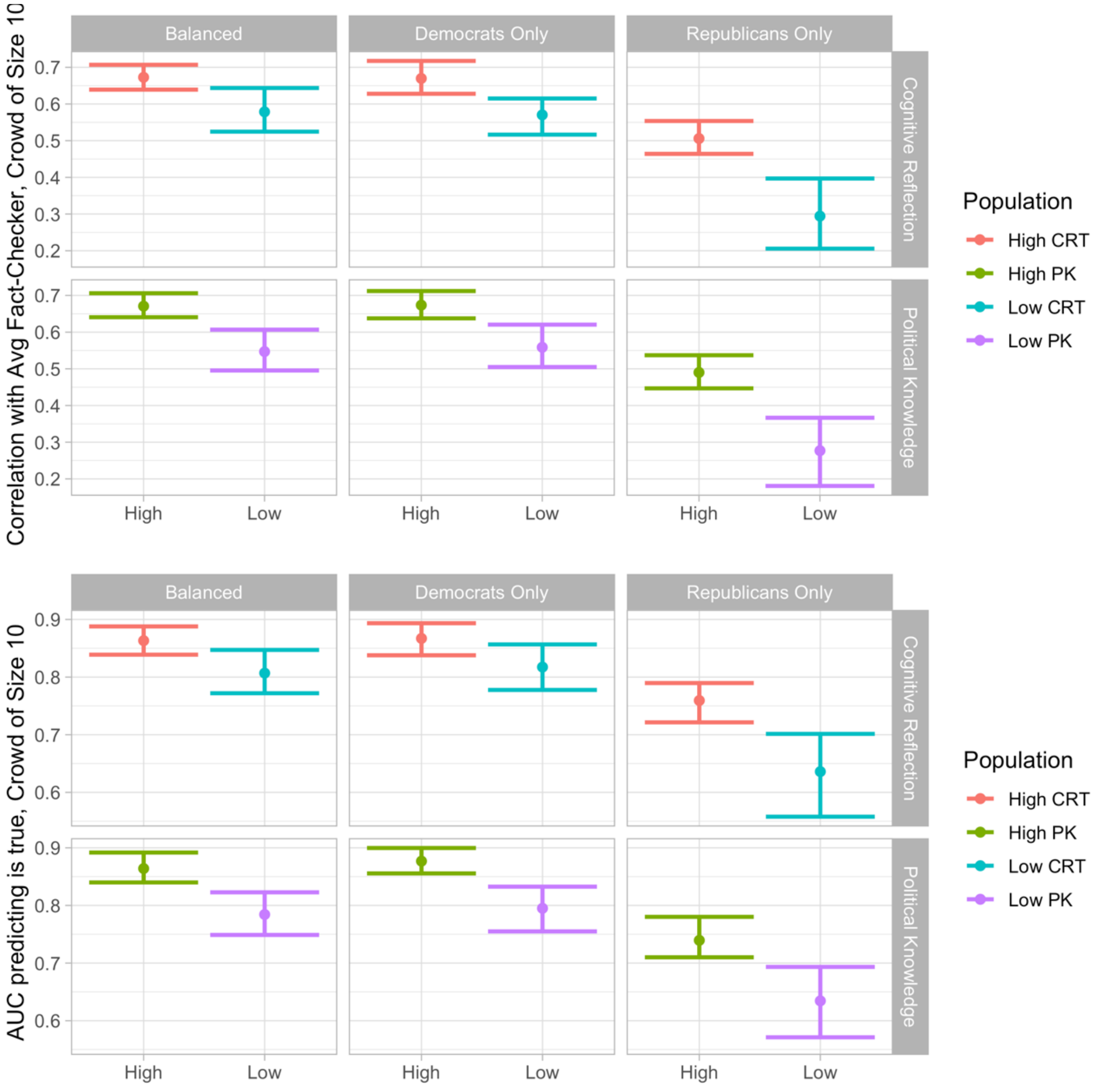

Figure S4. *Comparing the performance of high vs. low CRT and high vs. low political knowledge crowds for politically balanced, all Democrat, and all Republican populations, respectively. Panel A shows the correlation between the fact-checkers and a crowd of size 10, while Panel B shows the AUC of a model using the crowd's average continuous rating to predict the modal fact-checker categorical response with responses coded as 1 for True and 0 otherwise.*

8. Robustness: Performance as a Function of Crowd Size: Political Party, Cognitive Reflection, and Political Knowledge

In the main text Figure 3, we show the results of subsetting on partisanship, cognitive reflection, and political knowledge using a crowd of size 26, collapsing across the source and no source condition. Here we show performance as a function of *k*, the size of the layperson crowd. We use the identical procedure as described in Figure 3, but simulate the performance of crowds of size 2 through 26. As can be seen, (1) Democrat, (2) high CRT, and (3) high political knowledge crowds outperform the balanced crowd at small *k* values, but the advantage diminishes as the crowd size grows.

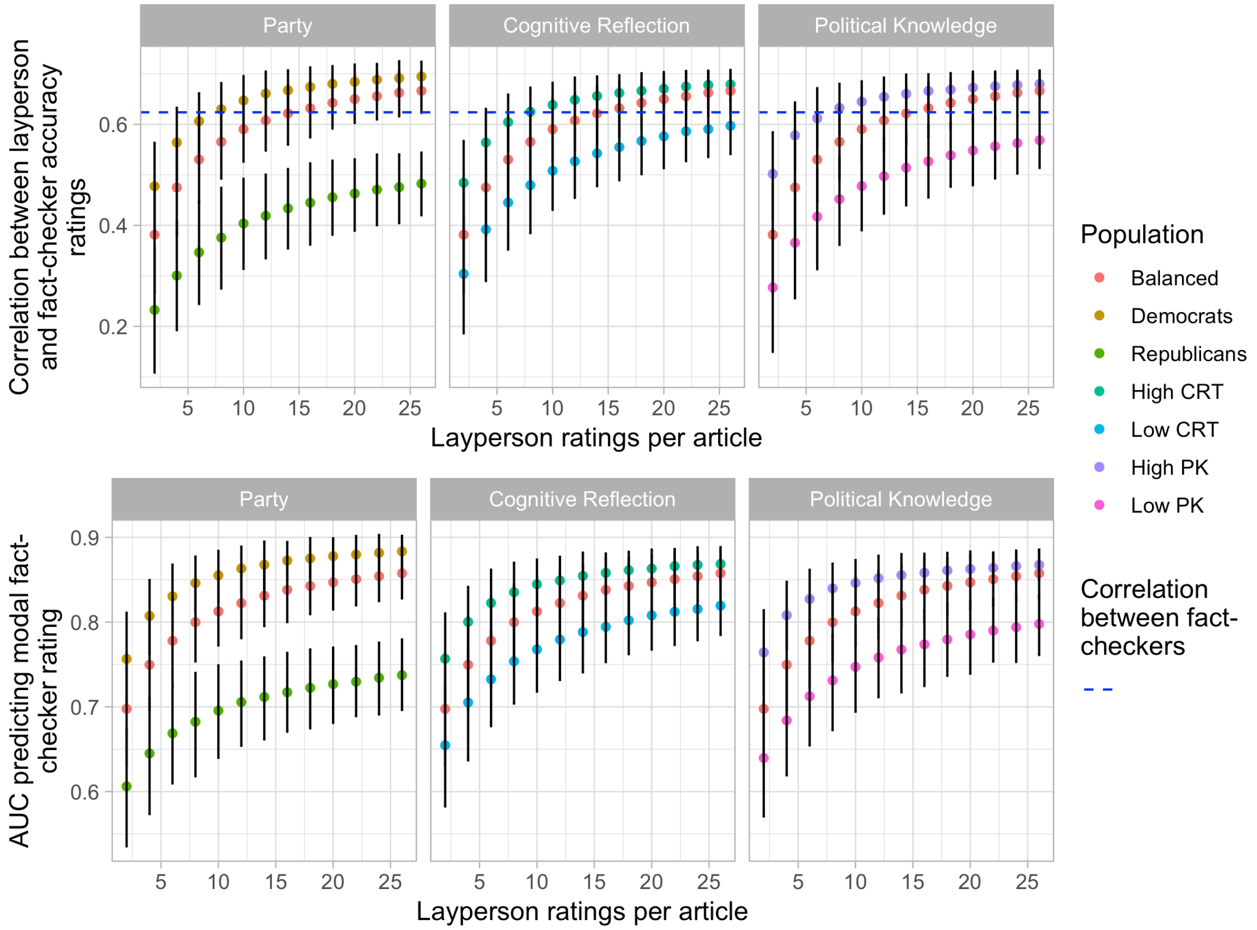

Figure S5. *(a-c) Correlation between average fact-checker rating and crowd and (d-f) AUC predicting modal fact-checker rating (1 = True, 0 = Not True) as a function of k, the number of layperson ratings per article. All panels compare performance to the baseline politically-balanced crowd, shown in red. Panels (a) and (d) show performance of an all Democrat vs. all Republican crowd. Panels (b) and (e) show performance for a politically-balanced crowd of participants who scored above the median on the CRT vs. at or below the median on the CRT; Panels (c) and (f) show a politically-balanced crowd of participants who score above the median on political knowledge vs. at or below the median on political knowledge.*

9. Robustness: Replicating Correlational Analysis with Alternate Set of Fact-checkers

To demonstrate that our results are not an artifact of the particular Upwork fact-checkers we used, and to support the validity of their ratings, we were also able to obtain ratings from a set of 4 journalist fact-checkers that a colleague had recruited to fact-check the same set of articles. These fact-checkers were professional journalists who had just completed a prestigious fellowship for mid-career journalists and had extensive experience reporting on U.S. politics. These journalists had an average inter-fact-checker correlation of .67 (similar to the .62 correlation among our Upwork fact-checkers), and the average of their ratings had a high degree of correlation with the average rating of our Upwork fact-checkers (r = .81). Furthermore, Figure S6 below shows that replicating our main analysis (Figure 1 of our paper) using the average rating from the 4 journalists (instead of the Upwork fact-checkers) yields qualitatively similar results. This demonstrates that our key result - that a relatively small number of laypeople can achieve similar correlation with the fact-checkers as the fact-checkers show with each other - is qualitatively robust to a different set of clearly qualified fact-checkers.

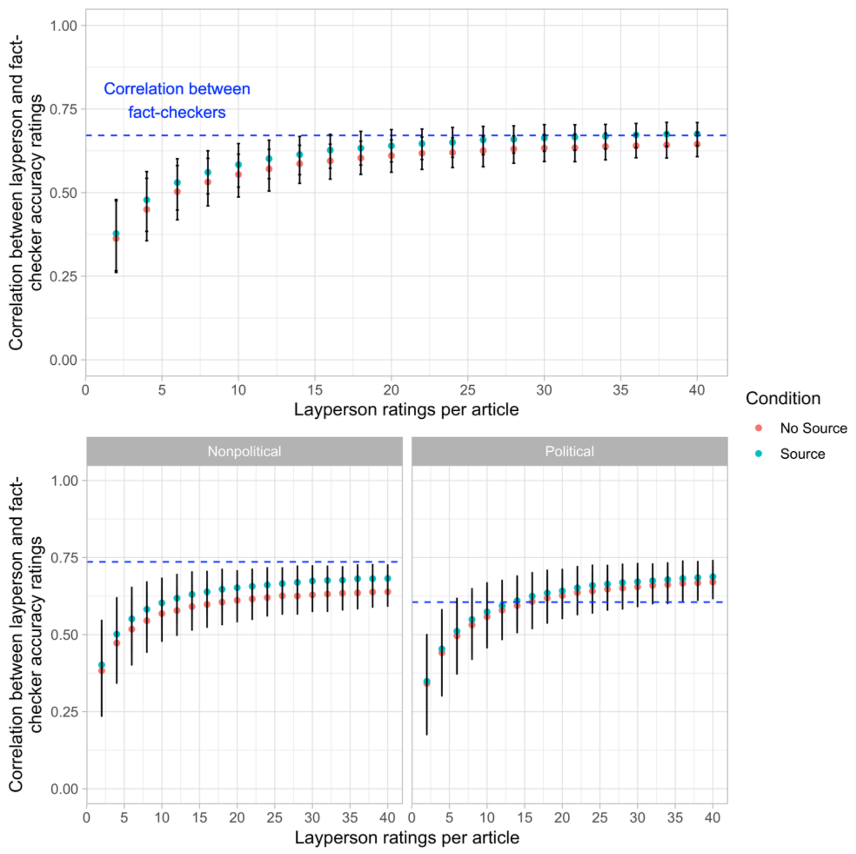

__Figure S6.__ Correlation across articles between (i) politically-balanced layperson aggregate accuracy ratings based on reading the headline and lede and (ii) average rating of 4 professional journalist fact-checkers' research-based accuracy ratings, as a function of the number of layperson ratings per article. Laypeople are grouped by condition (Source vs. No Source). Panels show results for (a) All articles, (b) Non-Political articles, (c) Political Articles. The dashed line indicates the average Pearson correlation between the fact-checkers.

10. Robustness: Replicating Correlational Analysis with Larger Crowds

We repeat the correlational analysis done in Figure 1 with crowds of size up to 100. As can be seen, while the correlation between fact-checkers and the crowd improves marginally after 25 responses, the performance largely asymptotes and does not improve at larger crowd sizes.

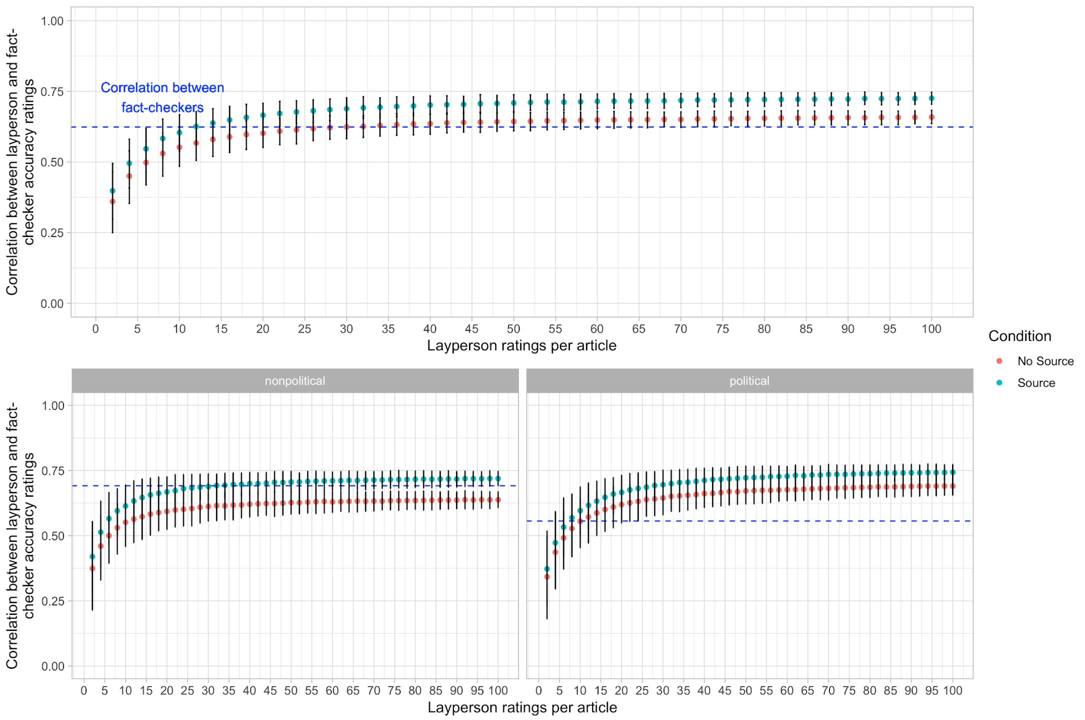

***Figure S7**: Correlation across articles between (i) politically-balanced layperson aggregate accuracy ratings based on reading the headline and lede and (ii) average fact-checker research-based aggregate accuracy ratings, as a function of the number of layperson ratings per article (up to 100). Laypeople are grouped by condition (Source vs. No Source). Panels show results for (a) All articles, (b) Non-Political articles, (c) Political Articles.*

11. Robustness: AUC Analysis, Excluding "Couldn't Be Determined" ratings

We believe that an article should only be classified as "true" if there is evidence in favor of being true - and therefore that "Couldn't Be Determined" counts against being "true". However, one could argue that "Couldn't Be Determined" ratings should be excluded from analysis since fact-checkers could not make a judgment for those articles. Therefore, we repeat our AUC analysis, excluding ratings of "Couldn't Be Determined" by the fact-checkers. The results are extremely similar to those presented in the main text, since only a handful of articles changed rating.

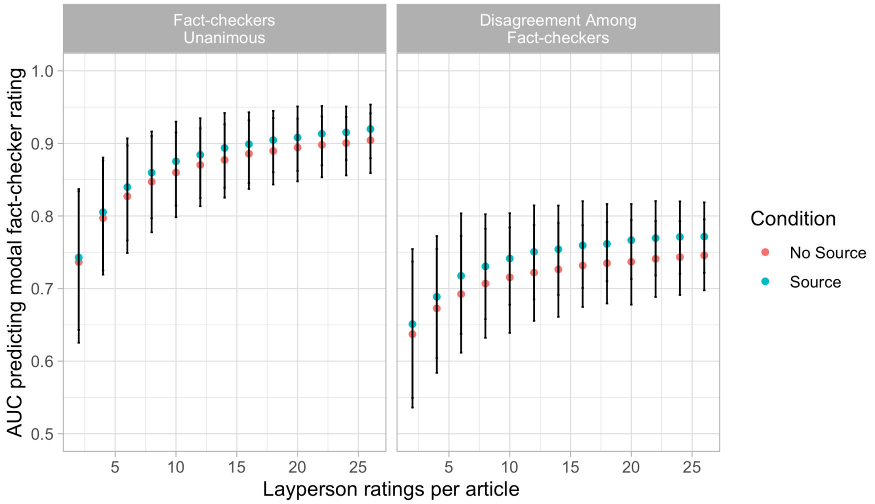

Figure S8: *Classifying articles as true versus non-true based on layperson aggregate Likert ratings. AUC scores as a function of the number of layperson ratings per article and source condition for articles with (a) unanimous fact-checker ratings and (b) non-unanimous ratings, excluding ratings of "Couldn't Be Determined".*

12. Robustness: Correlational Analysis with a Single Fact-Checker

One potential methodological concern is that we are comparing the correlation between *averages* of laypeople and fact-checkers to the average correlation between three *individual* fact-checkers. To address this potential measurement artefact, we show a plot with the correlation between the average crowd and a single fact-checker's ratings (rather than taking the average of all three fact-checkers). Note that the results are qualitatively similar to Figure 1 in our manuscript, although the number of laypeople it takes to match the fact-checker performance is higher than when averaging the three fact-checker responses.

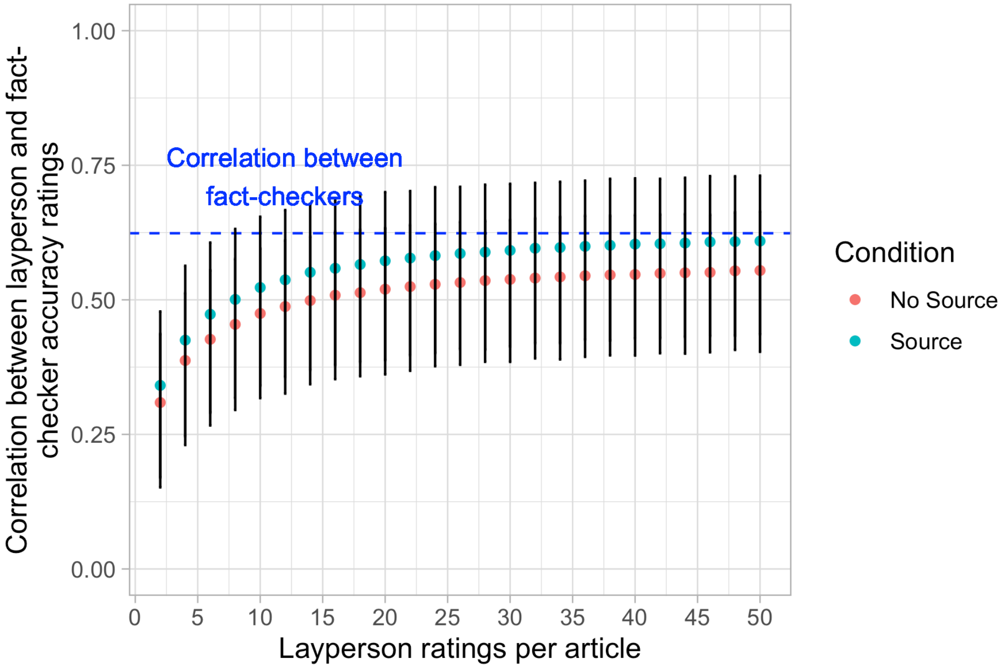

Figure S9: Average correlation across articles between (i) politically-balanced layperson aggregate accuracy ratings based on reading the headline and lede and (ii) single fact-checker, as a function of the number of layperson ratings per headline.

13. Robustness: Individual Differences for Political vs. Non-political headlines

We recreate the output in figure 3 examining the performance of crowds with different layperson characteristics for political and non-political headlines separately. We find similar results for political and non-political headlines, with Democrats outperforming Republicans, High CRT outperforming Low CRT, and High Political Knowledge outperforming Low Political Knowledge.

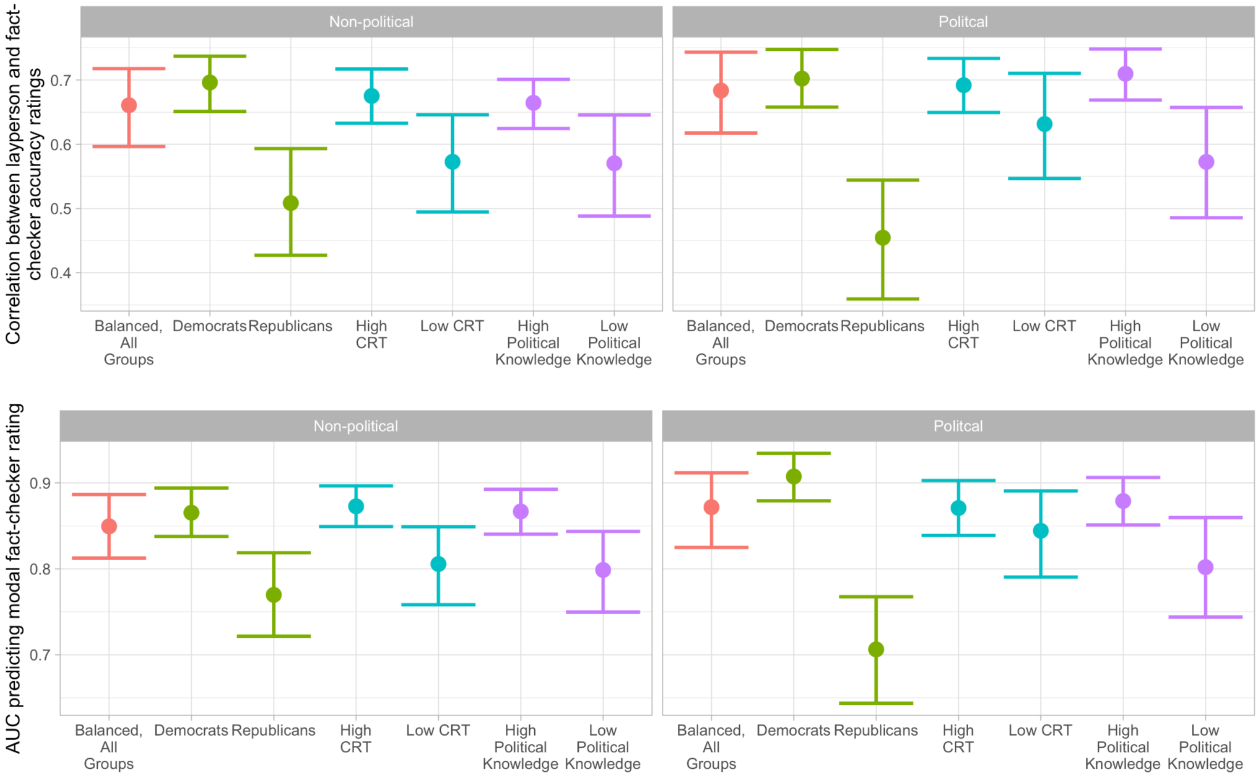

***Figure S10**: Comparing crowds with different layperson compositions to a baseline, politically-balanced crowd for non-political vs. political headlines. (a) (b) Pearson Correlations between the average aggregate accuracy rating of a crowd of size 26 and the average aggregate accuracy rating of the fact-checker for nonpolitical vs. political headlines, respectively. (c) (d) AUC for the average aggregate accuracy rating of a crowd of size 26 predicting whether the modal fact-checker categorical rating is "true" for nonpolitical vs. political headlines, respectively.*

14. ROC Curves for Predicting Fact-Checker Binary Ratings with Laypeople Continuous Ratings

We provide the ROC curves for our AUC analysis in which we used the crowd average Likert scale ratings to predict the fact-checker modal binary ratings (where "True" was coded as 1, else 0), annotated with the 1 - 7 Likert score cutoffs. While these curves are informative, we believe

they underscore our argument for using a continuous truth rating applied in newsfeed ranking rather than a binary cutoff.

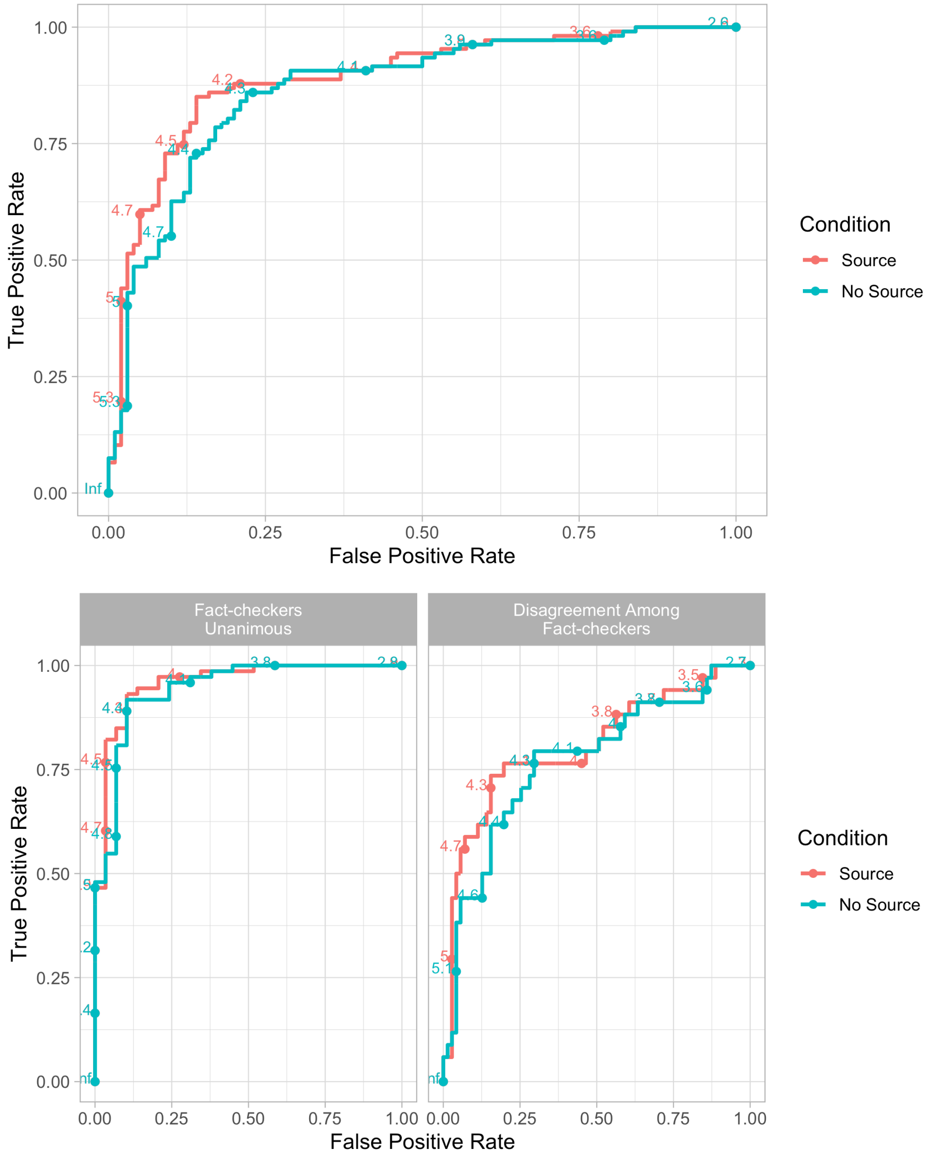

Figure S11: *ROC curves for a model that uses the average Likert rating of a crowd of size 26 to predict the fact-checker modal binary ratings (with "True" coded as 1, and all other answers coded as 0). Panel (a) shows the performance for the entire set of articles, and Panels (b) and (c) show performance for articles with unanimous and non-unanimous fact-checker agreement, respectively.*

15. Qualitative Examination of Disagreement Among Fact-Checkers

In this section, we provide more insight into the nature of the disagreement among the fact-checkers. In particular, we provide the categorical assessment, average accuracy rating, and rationale given by each fact-checker for 3 URLs from the set of 20 URLs that we had each fact-checker initially rate to show their competency. These 3 URLs are examples of where there was disagreement among the fact-checkers about whether or not the focal claim was

true. As part of the initial competency assessment, for URLs where there was disagreement, we gave the other fact-checkers' rationales to the fact-checker that disagreed with the modal assessment. Thus, we also include the disagreeing fact-checker's response as (sometimes lightly edited for conciseness). Our assessment of these texts (and the others in the initial set of 2) is that there was legitimate disagreement between the fact-checkers.

DOES ELIZABETH WARREN HAS STATISTICALLY LESS NATIVE AMERICAN BLOOD THAN AVERAGE WHITE AMERICAN?

Massachusetts Sen. Elizabeth Warren released the results from a DNA test Monday proving that she has statistically less Native American DNA than the average American white person.

https://dailycaller.com/2018/10/15/elizabeth-warren-less-native-american-dna/

FC1: Misleading; 3.57. The article's title was updated to reflect the updated information that came out, but the first sentence apparently wasn't updated. The entire article is a bit of a mess because of the updated information.

FC2: Misleading; 2.00. Nuances, nuances. All is much more complicated and even sources they cite are saying that. The article is pure clickbait article, however they have added an edit mentioning complexity of the whole thing. Problem is that the article is still showing a shallow understanding of it.

FC3: True; 6.71. It's nice that this piece has a correction as it should, and that helps with the trustworthiness and reliability scores. The only gripe I can offer is incidentally addressed by that correction: the distinction between heritage as calculated from generation versus ancestry by proportion of genetic material. The piece directs the reader on where to learn more about the nuances at play, but conflating the two remains reasonable in this situation as Elizabeth Warren's report did not provide the percentage figure for genetic material, necessitating a calculation of heritage based off of generations and the comparison of that to the distribution of genetic material you can find nationally. The nuances at play aren't an issue even in the absence of elaboration, but the piece even directs acknowledges the nuances and directs the reader as to where they can learn more about it.

FC3 response:

So the issue raised by the two other fact checkers in this case were that, regardless of correction, the article makes a false assertion that the DNA report Warren released proved she had less Native American genetic material than the average white American — this led to their classing of the article as misleading/false.

In my work on this article, I also honed in on the piece's claims regarding Warren's genetic material and heritage and how it relates to a subset of the population. There was three questions to answer here: (1) how much Native American genetic material does Warren have; (2) how much Native American heritage does Warren have; and (3) how does Warren's DNA and heritage compare to the white American subset.

DNA. The piece itself did not provide a figure for Warren's proportion of Native American genetic material. As a result, I had to seek such a figure in the DNA report, produced by one Dr. Bustamante, which Warren published. (See: http://templatelab.com/bustamante-report-2018/). Bustamante reported that: (1) at a 99% confidence level, about 95% of Warren's genetic material was identified as European; (2) the proportion of Warren's actual European genetic material was likely higher than 95%; (3) at a 99% confidence level, 5 DNA segments were identified as Native American, of a combined about 12,300,000 bases and about 25.6 centiMorgans; (4) at a 99% confidence level, the unassignable DNA segments were of a combined about 267,650,000 bases of about 366 centiMorgans; (5) a reference

human has 46 chromosomes in 23 pairs, with a combined about 6,469,660,000 bases and of about 7,190 centiMorgans. It should be noted that the number of bases would indicate the quantity of genetic material discussed, rather than measurements in centiMorgans indicating that. (See: https://www.genome.gov/genetics-glossary/Centimorgan). It should also be noted that the provided figures were all approximations and that the overall figure, itself an approximation, was for a reference human rather than for Warren as an individual as Warren's individual figure was not provided by Bustamante. Ultimately, we can calculate that Bustamante reported the proportion of Warren's genetic material assignable as Native American at the 99% confidence level as compared to a reference human at about 0.19%.

HERITAGE. 'Heritage' is a less firm term than 'proportion of genetic material'. For example, while heritage could refer to an individual's inherited genetic material, it could also refer to an individual's ancestors across preceding generations. In this way, and as discusses in the Washington Post article referenced by The Daily Caller, an individual's heritage in terms of ancestors and generations may differ from an individual's heritage in terms of inherited genetic material. For example, an individual with an unadmixed British father and an unadmixed Chinese mother, or a heritage in terms of ancestors of 50% Chinese, may in actuality only have 40% of their genetic material be assignable as Chinese as a result of biological processes. Additionally, it isn't possible to conclude with certainty just from analyzing an individual's genetic material when or how many times genetic material of a certain group, say Chinese genetic material, was introduced into that individual's lineage. This, again, is due to biological processes. Bustamante does, however, conclude from his analysis that an unadmixed Native American ancestor entered Warren's lineage about 8 generations, or between 6 and 10 generations, prior. Warren would have 64 ancestors in generation 6, 256 ancestors in generation 8, and 1024 ancestors in generation 10. Calculating heritage in terms of ancestors using Bustamante's conclusion, Warren's Native American heritage would therefore be as high as 1.562% but as low as 0.097%.

WHITES. There isn't even a supreme definition of who qualifies as 'white', let alone a supreme figure for how many white Americans there are or a supreme approximation of their genetic makeup. Bustamante's report never uses the word "white" but rather European, and The Daily Wire never elaborated on their use of the word "white" with a definition, except to reference a study which discusses "European Americans". The US Census Bureau, for example, operates with "white" defined as having ancestors from Europe, the Middle East, or North Africa."American", in contrast, can reasonably be assumed to mean individuals who are US citizens. Even making the assumption that those Americans referred to as "white" by The Daily Wire are those Americans with European ancestry, not all Americans self-identify as "white". As an example, even a 'black' or a 'Chinese' American with a known European ancestor, say Portuguese, from the distant past, say three centuries prior, may nonetheless decline to self-identify as 'white' in a legal, social, or research context. With this in mind, there moreover is not an abundance of research to indicate the proportion of Native American genetic material in the 'average white American'. The aforementioned study referenced by The Daily Wire provides an average proportion figure of Native American genetic material for the study participants who self-identified as European Americans of about 0.18%. However, the same study provides an average proportion figure of European genetic material for the study participants who self-identified as African of about 24%, underscoring that the 0.18% figure only corresponds to those who self-identified as European rather than those who were found to have European genetic material. (See: https://www.ncbi.nlm.nih.gov/pmc/articles/PMC4289685/pdf/main.pdf). Unfortunately, the study does not directly provide nor provide the data needed to calculate the proportion of Native American genetic material in the average American found to have European genetic material. As a result, Warren's here-calculated proportion of 0.19%. can't even fairly be compared to the study's proportion of 0.18%, which only describes individuals who self-identified as European and excludes those individuals who did not self-identify as European but who nonetheless were found to have European genetic material. On top of all that, the study does not define its participants as 'American' based on whether they are US citizens but rather based on where they live in the US.

The breakdown I've just provided demonstrates that while the Washington Post's clarification of heritage as defined by genetic material versus heritage as defined by ancestors was warranted, there is no evidence either in the Daily Wire article nor the Washington Post article nor in my own limited literature review to disprove the claim that "[Warren] has statistically less Native American DNA than the average American white person". Moreover, and even more importantly, that claim has so much wiggle room in how the terms "white", especially, and "American" may be defined that there isn't grounds to class it as false. Consider, moreover, that the central point of the article, which is that in the context of Warren's claims of Native American heritage she demonstrated herself to have a minuscule amount of Native American heritage, stands. Here we have a case where the study used to suposedly 'debunk' the claim is itself unsuitable to the task, the claim affords itself more than enough wiggle room anyways, and the central point of the article is left

standing. That's why, in good conscience, I can only class this piece as True while acknowledging its inaccuracies rather than class it as Misleading, much less False.

Toxic, treasonous media pushing "white supremacist" hoax and hit lists of Trump supporters in desperate scheme to drive America into civil war

It's now obvious the malicious, toxic media is pushing a "white supremacist" hoax in a desperate scheme to drive America into a civil war. The entire left-wing media has now become nothing more than a hate machine that's spreading its "daily hate" to radicalize left-wing Americans into an unprecedented level of hatred, insanity and violence.

https://www.naturalnews.com/2019-08-07-toxic-treasonous-media-pushing-white-supremacist-hoax.html

FC1: False; 1.29. There are parts of the truth in many of the points mentioned. Overall, the individual points are highly misleading and the overall article false.

FC2: False; 1.00. There are many different things in this article, many conspiracy theories and a lot of racism. I don't think it even deserves real debunking.

FC3: True; 3.86. My assesment of "true" for the piece isn't a confirmation of the opinion or analysis in this piece, but rather just of the facts provided and of the consistency between title and body: the opinion and conclusions may be criticized, but the promis of the title is delivered on and the facts provided are with one minor exception worthy of characterization as accurate. This piece doesn't seem to offer strict falsehoods or act in bad faith, but it is heavy with opinion, interpretation, and argument to an extreme degree— it's not a news piece, and it's certainly a biased piece. One of the central points of the article, for example, is that the media is pushing a hoax that mass shootings are motivated by white supremacy. The facts it provides in support of this claim are provided in good faith and appear to be produced through some due diligence. Consider the work of Daniel Greenfield with the David Horowitz Freedom Center who arrives at a figure showing that whites committed only a minority of mass shootings in 2019: he used an accepted pool of data, the product of a volunteer and crowdsourced endeavor, and a particular definition. A different definition, such as that used by Mother Jones, produces a very different figure contending the opposite, but both can be considered accurate given the data and criterea at hand and made in good faith. To make an accurate assessment, which no one has, a research project would have to be undertaken which surveys law enforcement across the country at the federal, state, and local level, a massive undertaking, and uses a particular definition for what's being considered. In short, I'm not prepared to call that evidence inaccurate or misleading. Another case in the piece which I'm not prepared to call inaccurate or misleading is where it claims that Joe Biden said he was going to send armed federal agents to confiscate guns: in the respective interview, he says that he's going to 'come for the guns' of those with assault weapons, then says he'll do so through a gun buyback program rather than through confiscation, then says he can't confiscate because there isn't a law on the book by which he can do so. Ultimately, there's reasonable wiggle room to interpret that in a scenario where there's a mandatory gun buy back program, for example, those who resist will ultimately face law enforcement. These kinds of incidents where true statements or incidents are heavily interpreted or argued to a far conclusion appear throughout the piece. Indeed, there are many opinions, or conclusions, throughout the piece which stand alone or aren't arrived at through evidence in the piece: take the conclusions that the media want America to fall into civil war and be destroyed, for example. Those aren't facts you can simply call correct or incorrect, rather they're matters of opinion, of political analysis by the author, and to whatever degree of rhetoric. The claim about a 'hit list' is similar, in that the interpretation is within the realm of rational arguement, but it has to be arrived at through interpretation. It's not misleading, but rather within the realm of analysis and not dependent on factual errors. There's only one incident I found of simple factual inaccuracy rather than a matter of heavy interpretation, opinion, or argument, and that's in the case of the 'death camp' posters, where the inaccuracy is that the posters were supposedly posted across New York City when actually they were posted in a Long Island town many miles away. This piece is characterized by heavy opinion, interpretation, and argument, and sometimes standalone conclusions or even calls to action. When it comes to the facts

featured and without making judgment on the opinion or analysis, with one relatively minor exception regarding the geographic distribution of posters, the facts themselves were accurate.

FC3 response to FC1 and FC2 rationales:

This piece was not an easy one to assess, and that should be apparent in light what a lengthy remark I provided with my assessment of it.

[Furthermore] the piece does not claim that 'white supremacy' is a hoax — there's nothing in the piece to suggest a claim that there aren't white supremacists — rather the claim of the piece is that whereas the media is perpetrating a hoax that white supremacy is driving mass shootings, the media's claim is untrue. The piece goes on to support that point with evidence. I won't make a judgment on the opinion or the analysis, but I don't have any significant factual errors to point to. There is also the question of whether the piece's claim that the media is trying to drive the country to civil war is a conspiracy theory. I judge such claims in the piece to be rhetoric or analysis, and outside of my mandate except counting towards the piece's low scores in Bias, Subjectivity, and Trustworthiness. Had the piece included falsehoods as evidence to back up such 'conspiracy theory' claims, I may have had grounds to class this piece as False, but where the piece goes so far as that it does not bring falsehoods with it. I take care to exclude emotion from these assessments, and neither opinion nor analysis are grounds for classing this piece as False in the absence of material inaccuracies. Moreover, I cannot class this piece as Misleading when it delivers exactly what it says on the tin and doesn't equivocate et cetera. The piece doesn't pretend to be something it's not and doesn't cross any ethical lines. When you cut out the emotional knee-jerk, separate the fat, which is the analysis and opinion, from the meat, which is the factual claims, as much as I can do is to class the piece as True while marking it rock-bottom for Bias, Subjectivity, and Trustworthiness. I stand by my classification.

Ginsburg Can't Remember 14th Amendment, Gets Pocket Constitution from the Audience

Some of our Supreme Court justices care more about politics and logical gymnastics than the text of the Constitution.

https://conventionofstates.com/news/ginsburg-can-t-remember-14th-amendment-gets-pocket-constitution-from-the-audience

F1: Misleading; 3.14. The article's title is misleading, while the actual article is correct, although written in an biased manner. From the video, it doesn't appear Ginsburg forgot the amendment so much as she wanted to cite it correctly.

F2: Misleading; 3.00. Justice Ruth Bader Ginsburg indeed needed a text of Constitution in front of her eyes, however it is probably more related to her health state, age and the point she was making. She definitely knows the subject well as later on during the same conversation she was referring multiple times to the 14th amendment. Besides that she has been giving lectures to high school students on this very topic. So the article is very biased, uses the event to ridicule the Judge and has call to action just under the story.

F3: True; 5.29. Factually true, but of course biased and even calling to action. It didn't clarify that she wanted to find her pocket constitution to have on hand before she began answering the question and that she clearly knew where she was going before she got her hands on one, and the title is rather sensational when really it's more fair to her to say that she couldn't remember the text verbatim, but I can't call this inaccurate simply for being unflattering. Furthermore, that the body and truth delivers on the title makes it all the harder to call this misleading — I wouldn't call it misleading, but it could give a kinder context.

FC3 response:

This particular assessment was not an easy one to make, and the reasons why are echoed in the remarks of the other fact checkers. I ultimately had to class this piece as either Misleading or True because while containing no factual errors, the piece was sensational, biased, and deeply unflattering. It's fair to say that this title is sensational and unflattering, but it is factually true and the context is provided in the article. I'm understanding of the classifications as Misleading, because I too had to reconcile the sensational hook with the factual accuracy, but ultimately the article provides the context and delivers on what it sets out to. I can't class a piece as misleading simply for being unkind, unflattering, charged, or sensational — that would be an inappropriate, emotionally-driven judgment rather than an ethical and objective one. I understand though respectfully disagree with those who classed it as Misleading. I stand by my classification of the piece as True though it wasn't the easiest decision to reach.

16. Generalization to a Researcher-Selected Dataset

We also applied the same crowdsourcing approach to an alternative dataset from Pennycook & Rand (2019) in order to explore the generalizability of our findings to different stimulus sets. The alternative dataset contained 30 researcher-selected headlines, half true and half false, balanced between pro-Democrat, pro-Republican, and neutral. The crowd was composed of 800 participants recruited from Amazon Mechanical Turk. We used the same methodology as in Figure 2 of the main text to use the crowd's ratings to classify headlines as True or Not True. We find substantively similar findings to those presented in the main text, with an AUC of .95 (95% CI: [.85,1]) for a crowd of size 26. In fact, crowd performance was better on the researcher-selected stimulus set than the Facebook-provided one, suggesting that the set of headlines provided to us by Facebook was substantially more difficult to rate.

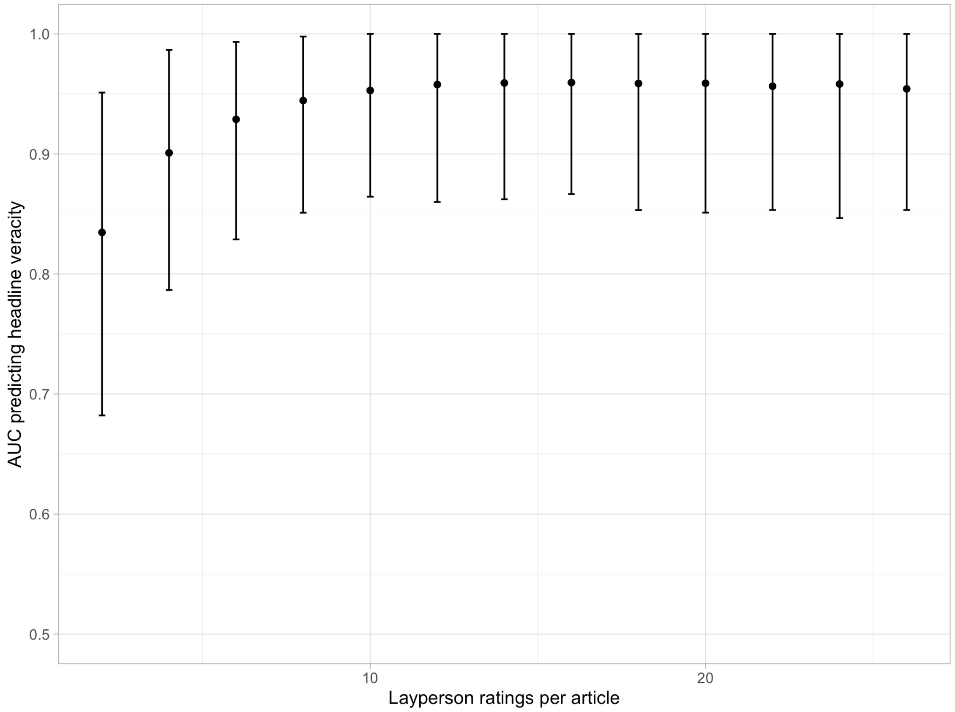

Figure S12: *AUROC for a model that uses the average Likert rating of a politically-balanced crowd to predict fact-checker ratings of "True" (1) vs. "Not True" (0).*
